# Supplementary material for: Reducing dementia-related stigma and discrimination among community health workers in Brazil: protocol for a randomised controlled feasibility trial
Source: BMJ Open. 2022 Jul 19;12(7):e060033. doi: 10.1136/bmjopen-2021-060033 (PMC9301803; doi:10.1136/bmjopen-2021-060033)
Supplement: Supplementary data [file bmjopen-2021-060033supp002.pdf]

Supplementary material II

Baseline and post-study assessment

Sociodemographic and work-related data

1. Date of birth: _____________
2. Age: _______
3. Sex: Female ( ) Male ( ) Prefer not to say ( )
4. School attendance:
   Primary school ( ) Middle school ( ) Undergraduate degree or above ( )
5. Religion: None ( ) Catholic ( ) Evangelical ( ) Other: _________________
   Prefer not to say ( )
6. Race: Black ( ) White ( ) Mixed-race ( ) Yellow ( ) Prefer not to say ( )
7. PCU name: ____
8. Number of months\years working as a CHW ___________________________
9. Do you have any personal experience working with people living with dementia or carers?
   Yes ( ) No ( ) Does not know or cannot remember ( )
10. Do you have any professional experience working with people living with dementia or carers?
    Yes ( ) No ( ) Does not know ( ) Cannot remember ( )
11. Have you ever had any training on dementia?
    Yes ( ) No ( ) Does not know ( ) Cannot remember ( )
12. If you said yes to the question above, when was this training and what was the focus?
    Month and year: __________________________
    Topic: ____

Global Survey on Stigma and Dementia (2019)

Questions on knowledge, attitudes and intended behaviours related to people living with dementia. Items available upon request: https://www.alzint.org/resource/world-alzheimer-report-2019/

Feasibility

Satisfaction with the intervention (Wang et al., 2017)

Instruction: Please read each of the items below and circle the answer that best reflects your opinion. We are trying to improve the intervention you participated in, so please be as honest as possible with your answers so we can do better in the future.

Questions	Strongly disagree	Disagree	I neither agree nor disagree	Agree	Strongly agree
1. Before participating in the program, my knowledge about dementia and caring for people living dementia was very limited.	1	2	3	4	5
2. This program has helped me learn new knowledge on dementia and care for people living with dementia.	1	2	3	4	5
3. The activities were necessary and useful.	1	2	3	4	5
4. The discussions improved my learning in relation to people living with dementia.	1	2	3	4	5
5. Discussions on how to translate knowledge into practice helped me think about how to apply knowledge in my practice.	1	2	3	4	5
6. The materials were clear and informative.	1	2	3	4	5
7. Overall, the program has reached my expectations.	1	2	3	4	5
8. Overall, I believe the program will help improve my work with people living with dementia and their carers.	1	2	3	4	5

Open questions

Please answer the questions below with your opinions about your participation in this project. We are trying to improve the intervention you participated in, so please be as honest as possible with your answers so we can do better in the future.

1. Would you recommend this program for another CHW? Why?

2. Overall, from 0 to 10, how satisfied were you with your participation in the program? Why? (0- no satisfied at all, 10- very satisfied)

3. What did you like the most in this programme? Why?

4. What did you not like in this programme? Why?

5. Was there anything that got in the way of your participation? If so, what was it?

6. What\How could the program be improved?

7. Have you talked about any of the aspects discussed in this programme with anyone?

8. How do you think participation in this program may have affected or will affect your practice as CHW?

9. Could you give a concrete example of any change that has already occurred in your CHW's activities in relation to people living with dementia and their families after your participation in the program? If you believe there has been no change, answer "no."
