# Supplementary material for: Reducing dementia-related stigma and discrimination among community health workers in Brazil: protocol for a randomised controlled feasibility trial
Source: BMJ Open. 2022 Jul 19;12(7):e060033. doi: 10.1136/bmjopen-2021-060033 (PMC9301803; doi:10.1136/bmjopen-2021-060033)
Supplement: Supplementary data [file bmjopen-2021-060033supp003.pdf]

# Supplementary material III

## Interview schedule

**Introduction**

**1. Thank the participant for taking part**

**2. The purpose of the interview**

I'm part of a project called STRiDE. The idea of the project is …

We would like to hear your ideas and suggestions related to your participation in this project, so I'm going to ask you a few questions about this. I have here a list of questions and topics that will serve as a general guide on what I would like to talk to you about today, but this is flexible since the most important thing is that we talk about what is important to you.

The duration of the interview depends on your availability and interest. It usually lasts 60 min, but we can use more or less time. You can stop the interview at any time if you don't want to continue. It's okay if you want to take a break when you feel the need.

**3. Reminder of ethical issues related to consent, anonymity, confidentiality, and safety measures**

**4. Presentations**

**5. Questions**

Is there anything else you'd like to know about your participation or any other questions you may have?

**6. Interview**

1. Why did you decide to participate in this study?
2. What do you remember the most of what we covered in the sessions?
3. How would you describe the training and key messages covered?
4. Could tell me about any learning points you had through this programme?
5. What did you like the most and what did you like the least about this program?
6. Do you think the duration of the sessions and the whole study were sufficient or should be shorter or longer? Why is that?
7. If you could participate again, what would you like to be different?
8. What do you think we could do to get as many CHWs as possible involved in activities like this?
9. Do you consider that your participation in the programme is likely to change your practice as a CHW with people living with dementia and their carers in any way? Why\How?
10. Do you think participating in this programme and applying what you've learned in practice can help improve the care provided for people living with dementia and their carers in any way in real life? Why\How?
11. In your opinion, is there anything you think would limit how much you can put in practice what you learned during the intervention? Could you explain why?

12. In relation to the previous question, how could we researchers mitigate such factors?
13. Would you recommend this program to someone? Why is that?
14. Do you have any other comments to make about the intervention?

**End of interview**

1. Remind the participant about the end of the interview.
2. Thank the participant for their valuable contributions.
3. Tell the participant what will happen now (e.g. how the data will be used).
