# Supplementary material for: Reducing dementia-related stigma and discrimination among community health workers in Brazil: protocol for a randomised controlled feasibility trial
Source: BMJ Open. 2022 Jul 19;12(7):e060033. doi: 10.1136/bmjopen-2021-060033 (PMC9301803; doi:10.1136/bmjopen-2021-060033)
Supplement: Author's manuscript [file bmjopen-2021-060033.draft_revisions.pdf]

BMJ Open is committed to open peer review. As part of this commitment we make the peer review history of every article we publish publicly available.

When an article is published we post the peer reviewers' comments and the authors' responses online. We also post the versions of the paper that were used during peer review. These are the versions that the peer review comments apply to.

The versions of the paper that follow are the versions that were submitted during the peer review process. They are not the versions of record or the final published versions. They should not be cited or distributed as the published version of this manuscript.

BMJ Open is an open access journal and the full, final, typeset and author-corrected version of record of the manuscript is available on our site with no access controls, subscription charges or pay-per-view fees (http://bmjopen.bmj.com).

If you have any questions on BMJ Open's open peer review process please email info.bmjopen@bmj.com

BMJ Open

Reducing dementia-related stigma and discrimination amongst community health workers in Brazil: Protocol for a randomized controlled feasibility trial

Journal:	*BMJ Open*
Manuscript ID	bmjopen-2021-060033
Article Type:	Protocol
Date Submitted by the Author:	09-Dec-2021
Complete List of Authors:	Oliveira, Deborah; Universidade Federal de São Paulo, School of Medicine, Department of Psychiatry<br>Godoy, Carolina; Universidade Federal de São Paulo, School of Medicine, Department of Psychiatry<br>da Mata, Fabiana ; Universidade Federal de São Paulo, School of Medicine, Department of Psychiatry<br>Mateus, Elaine; Brazilian Federation of Alzheimer's Associations; Universidade Estadual de Londrina (UEL), Applied Linguistics<br>Franzon, Ana Carolina; Brazilian Federation of Alzheimer's Associations<br>Farina, Nicolas; Brighton and Sussex Medical School, Centre for Dementia Studies<br>Evans-Lacko, Sara; London School of Economics and Political Science, Personal Social Services Research Unit<br>Ferri, Cleusa; Universidade Federal de Sao Paulo, School of Medicine, Department of Psychiatry; Hospital Alemão Oswaldo Cruz, Health Technology Assessment Unit
Keywords:	Dementia < NEUROLOGY, GERIATRIC MEDICINE, MENTAL HEALTH, PRIMARY CARE, Delirium & cognitive disorders < PSYCHIATRY, Old age psychiatry < PSYCHIATRY

SCHOLARONE™
Manuscripts

Reducing dementia-related stigma and discrimination amongst community health workers in Brazil: Protocol for a randomized controlled feasibility trial

Authors: Déborah Oliveira[1], Carolina Godoy[1], Fabiana A. F. da Mata[1], Elaine Mateus[2,3], Ana Carolina Arruda Franzon[2], Nicolas Farina[4], Sara Evans-Lacko[5], Cleusa P. Ferri[1,6]

Affiliations:

1. Universidade Federal de São Paulo (UNIFESP), School of Medicine, Department of Psychiatry. São Paulo, Brazil.
2. Brazilian Federation of Alzheimer's Associations (FEBRAZ). Paraná, Brazil.
3. Universidade Estadual de Londrina (UEL), Applied Linguistics, Department of Modern Languages, Paraná, Brazil.
4. Brighton and Sussex Medical School, Centre for Dementia Studies, Brighton, United Kingdom.
5. London School of Economics and Political Science, Care Policy and Evaluation Centre, London, United Kingdom.
6. Hospital Alemão Oswaldo Cruz, Health Technology Assessment Unit, São Paulo, Brazil.

Corresponding author: Déborah Oliveira. Universidade Federal de São Paulo (UNIFESP), School of Medicine, Department of Psychiatry. Rua Major Maragliano, 241, Vila Mariana, Post Code 04017-030, São Paulo, Brazil. E-mail: oliveiradc.phd@gmail.com

Word count: 3,494

Abstract

Introduction: Stigma and discrimination amongst healthcare workers can hinder diagnosis and the provision of appropriate care in dementia. This study is aimed at developing, delivering, and evaluating the feasibility of a group anti-stigma intervention to improve knowledge, attitudes, and behaviours in relation to people living with dementia among Community Health Workers (CHWs). **Methods and analysis:** This will be a randomized controlled feasibility trial conducted with 150 CHWs from 14 Primary Care Units (PCUs) in São Paulo, Brazil. PCUs will be randomly allocated (1:1) in two parallel groups - experimental group or control group. Participants from PCUs allocated to the experimental group will receive a group intervention involving audio-visual and printed materials, as well as elements of social contact. The intervention consists of three group meetings, held on three consecutive days. The control group will follow their usual routine. Knowledge, attitude and intended behaviour stigma-based outcomes will be assessed at baseline and at follow

For peer review only - http://bmjopen.bmj.com/site/about/guidelines.xhtml

up to both groups, with additional questions on feasibility for the experimental group at follow up. Around 10-15 participants will take part in follow up semi-structured interviews to further feasibility. Quantitative analyses will follow an “intention to treat” approach. Qualitative data will be analysed using content analysis. **Ethics and dissemination:** This study was approved by the National Commission for Ethics in Research in Brazil (*number*). Every participant will sign a consent form. Results will be disseminated through academic journals and events related to dementia. The intervention materials will be made available online.

Keywords: dementia; stigma; feasibility studies; primary care; community health workers

Strengths and limitations of this study

- This is the first study to develop, deliver and evaluate a controlled feasibility study of a group anti-stigma intervention to improve knowledge, attitudes, and behaviours in relation to people living with dementia among community health workers in Brazil.
- This study will seek to involve all the community health workers of all the primary care units of a large urban city in São Paulo, Brazil.
- The study includes quantitative and qualitative components that will assess the feasibility of the intervention from different perspectives and will include a follow up assessment which will help us understand any potential long-term impact.
- As this will be a feasibility study, no conclusions can be drawn about the effectiveness of the intervention, however we hope the information collected will help build a robust future randomized controlled trial.

Background

There are approximately 55.2 million people living with dementia worldwide and nearly 70% of this population live in low- and middle-income countries (LMICs), such as Brazil.[1] By 2050, this number is expected to increase to 139 million.[1] Stigma and discrimination related to dementia are common, which is particularly important among healthcare workers, considering the detrimental impact on the lives of those living with the condition and their families, such as by hindering access to diagnosis and support.[2,3] Combating the stigma related to dementia is a global health priority.[1]

Stigma occurs when a label associated with a negative stereotype is attributed to an individual characteristic, causing people with such characteristics to be seen as separate and of lower status compared to people without the characteristic.[4] When stigma occurs, 'power' is exercised by stigmatizers to keep stigmatized groups 'down' or

For peer review only - http://bmjopen.bmj.com/site/about/guidelines.xhtml

For peer review only

dominated/exploited; 'within', to maintain social norms; and 'away' by means of social exclusion.[5] In dementia, stigmatization occurs through negative stereotypes, related to cognitive decline can lead to depersonalization and considering the person as unable to continue to live in and contribute to society.[6–9] Although not every person living with dementia is an older person, older people living with dementia are likely to experience stigma related to dementia, as well as from ageism and ableism, further impacting their rights and wellbeing.[10]

Several studies provide robust evidence on the negative impact that stigma and discrimination has on people living with mental disorders more broadly.[11–13] Although there is a paucity of research on the impact of stigma on people living with dementia,[6] the existing evidence shows that stigma can lead to negative feelings about one self, shame, symptom and diagnosis concealment, negative social interactions, reduced access to care networks and social participation, and even suicide. [2,6,9,14–16] A global survey on attitudes towards dementia among members of the public, people living with dementia, family carers and healthcare professionals from 155 countries showed that over 85% of respondents living with dementia had their opinion not taken seriously, as well as between 35% and 57% (in high-income countries and LMICs, respectively) had been treated unfairly in intimate relationships. Moreover 40% of the general public believed health professionals ignore people living with dementia; and 35% of carers reported hiding the diagnosis of dementia of a family member.[2]

Limited knowledge about dementia, as well as widespread misbeliefs (e.g. believing that dementia is a natural part of ageing) and negative attitudes (e.g. considering people living with dementia are burdensome) may make health professionals less likely to detect dementia and provide adequate care to people affected by this condition.[3,9] In over 75% of cases globally, diagnosis of dementia is either not made, or is made at a later stage, when the person living with the condition is no longer able to make decisions about their life and wellbeing independently.[3] Structural forms of stigma – such as when systems, policies, or services are designed in a way that discriminates directly or indirectly against people living with dementia[17] – also contributes to poor healthcare provision in dementia, by means of lack of investment in appropriate services and in training for health and social care providers, who continue to be unprepared to diagnose and to provide care and support for people living with dementia,[3] further contributing to the experiences of stigma and discrimination experienced by these individuals. The current state of healthcare systems and providers can therefore be seen as a consequence, as well as a source of, stigma related to dementia.

For peer review only - http://bmjopen.bmj.com/site/about/guidelines.xhtml

A recent systematic review showed that most interventions to reduce mental health related stigma in LMICs are effective.[18] The most common approach is education; however, a wide variety of methods – from creative arts-based approaches to those which emphasise empowering people with mental health conditions – can be successful, including social contact interventions.[18] Currently, very little is known about interventions which reduce stigma related to dementia[19] and research on knowledge and attitudes related to dementia in Latin American countries is scarce.[20,21] There is an urgent need for further research on dementia-related stigma in different contexts and cultures.[1,6]

It has been estimated that 77% of people living with dementia in Brazil are not diagnosed.[22] Prevalence estimates of people living with dementia vary from 5.1% to 19.0% among those aged 65 and over.[23] Brazil still lacks well-funded and well-equipped health systems to meet the needs of the growing population of people living with dementia and their families. The country has a universal health system in which Primary Care Units (PCUs) are the first point of access for people who experience any physical or mental health problem. In most regions, PCUs have Community Health Workers (CHWs) who contribute to health promotion and disease prevention activities.[24] Most CHWs live in the same communities where they work, being a valuable point of contact and an intersection between health services and their communities. Health systems with CHWs have the potential to help expand the delivery of mental health care and close the existing mental health gap in LMICs.[25] However, the training activities offered to CHWs in Brazil is focused on the control of communicable diseases, maternal and child health and non-communicable physical diseases, such as diabetes and hypertension, with very little training dedicated to dementia. Providing CHWs with adequate knowledge on dementia, as well as on common stigmatising and discriminatory practices, and positive attitudes and behaviours towards people living with dementia may help increase the number of people living with dementia identified by the PCU, as well as the quality of the health care and support provided to these individuals.

Aim and objectives

The overarching aim is to design an anti-stigma intervention to reduce stigma and discrimination towards people living with dementia among CHWs in the city of São José dos Campos, Sao Paulo, Brazil and to pilot the intervention using a feasibility randomized controlled trial. The specific objectives are:

1. To develop a group-based intervention to improve knowledge about dementia, as well as attitudes and behaviours towards people living with dementia; and

For peer review only - http://bmjopen.bmj.com/site/about/guidelines.xhtml

2. To test the intervention in a randomized controlled feasibility study and evaluate the acceptability and feasibility of performing such an intervention in a future randomized controlled trial.

Methods and analysis

Study design

A randomized controlled feasibility study will be conducted as part of a large multinational research programme (*name omitted for peer review*) which aims to contribute to the improvement of care systems, treatment, and support for people living with dementia and their families in Brazil and other LMICs. An exploratory study was conducted previously involving semi-structured interviews and focus groups with people living with dementia, family carers, healthcare workers, and members of the public in three large cities in the state of Sao Paulo.[9,26] The findings from the study helped inform the content and design of this intervention.

Participants and sampling

All CHWs in each of the 20 eligible PCUs located in São José dos Campos will be invited to take part. Individuals working as a CHWs for less than three months will be excluded. Each PCU has approximately from 8 to 12 CHWs, totalling 160 to 240 potential participants. A proportion (30%) of refusals to participate and dropouts are expected (e.g., change in work schedules), therefore we estimate to include approximately 14 PCUs involving around 150 participants in the trial.

Method of generating the allocation sequence

Randomization and allocation will be conducted by blinded member of the research team at the PCU level. The PCUs whose managers agree to participate will be randomly allocated in two parallel groups – experimental group or control group – so that all CHWs of a given PCU who voluntarily accept to take part can be allocated in the same group forming a cluster and reducing the potential for contamination (e.g., sharing knowledge learnt in the intervention between PCUs). The allocation will be made randomly at 1:1 so that there is the same number of PCUs in each group, offering as much as possible balanced participation among CHWs from PCUs located in the various geographical regions, proportional to the total number of PCUs of each region (20%: north, 20%: south, 20%: east, 20%: west, 20%: centre). It will also be taken into account, as far as possible, balance between the characteristics of the participants (e.g. age, gender, time working as CHW).

For peer review only - http://bmjopen.bmj.com/site/about/guidelines.xhtml

Before informing the PCUs about the group to which their CHWs have been allocated, every CHW wishing to participate will read and sign a consent form. Recruitment and enrolment will be conducted by a separate member of the team, who will be blinded to the allocation. Then, all participants will be invited to complete the baseline measures (t1). The experimental group will be informed about the dates and timings of the intervention and the control group will be told that they will be allocated to a waiting list. After the completion of the final assessments from both experimental group and control group (t2), the intervention will be offered to the control group.

Development of the content of the intervention

Participants within the experimental group will receive a group intervention created by the researchers – DO (Nurse, Brazil), CG (Gerontologist, Brazil), CPF (Psychiatrist and Epidemiologist, Brazil), FAFM (Physiotherapist, Brazil), EM (Linguist, Brazil), ACAF (Journalist, Brazil), NF (Psychologist and dementia researcher, England), and SE-L (Stigma expert, England). The researchers have extensive experience in research and clinical practice with people living with dementia, CHWs and family carers. The intervention materials were informed by a rapid scoping review conducted by the team on dementia-related stigma in Latin America and the Caribbean (unpublished) and on the exploratory qualitative work conducted previously with people living with dementia, family carers, healthcare providers, and members of the public in Brazil.[9,26] This was also supplemented with the literature on ethical and patient-centred care for people living with some type of mental and/or neuropsychiatric disorder,[27] literature in the area of person-centred care for people living with dementia,[28–31] as well as by evidence for effective interventions to reduce stigma in LMICs.[11,13,18]

The researchers will use audio-visual and printed materials containing, for example, videos of people with dementia and carers sharing their personal experiences, reflexive activities, group discussions, and presentations through Power Point® (Table 1).

For peer review only - http://bmjopen.bmj.com/site/about/guidelines.xhtml

Table 1. Summary of the intervention components and activities

When		Why: what we want to achieve	What: topic covered	How: method or strategy
**Day 1:** Building knowledge and demystifying dementia: beginning the transformation process.	Session 1	• To understand the general structure of the intervention and get to know one another, which will be important to build a non-judgmental space of trust among the individuals and for their spontaneous contribution to the activities.<br>• To get in touch with individual and shared beliefs and questions related to dementia, and to gain a better understanding of the condition and of the 'individual behind the disease'.	• What is dementia<br>• What is not dementia<br>• How people living with dementia are seen.	• Before the session starts, we ask participants to individually write down their views on dementia and people living with dementia using directed questions.<br>• We provide an interactive presentation on knowledge and common beliefs related to dementia.<br>• Each individual reads their views and shares with the group voluntarily. The group reflects and discusses these based on the presentation delivered earlier.
	Session 2	• To understand what dementia is, what dementia is not, and what treatment and care possibilities exist.<br>• To understand how dementia can affect people living with dementia and their families.<br>• To experience, through group dynamics, the impacts caused by prejudice, discrimination, and negative language on the lives of people living with dementia.	• What is dementia<br>• What is not dementia<br>• Known and unknown, modifiable and non-modifiable risk factors.<br>• Pharmacological and non-pharmacological interventions can help people who live with dementia and their carers to have quality of life.<br>• Management and control of dementia symptoms.	• This will be a dynamic session in which we ask questions to the group in one slide, and provide feedback in the following slide, and so on.<br>• After, we will have the "secret box" dynamic: we will distribute individual boxes containing two photos of famous people and a mirror at the end. We ask participants to describe the characteristics of the two people and then to describe themselves. We try to identify differences in how they would describe 'others' and how they would describe 'themselves' to stimulate empathetic and non-judgemental approaches.<br>• We will hold a reflective and introductory debate on stigma, prejudice, discrimination, and use of language.<br>• We will promote reflection on how the participants felt when placing themselves in the 'shoes' of someone living with dementia.
**Day 2:** Breaking down labels and stereotypes and improving attitudes towards people living with dementia.	Session 1	• To understand how thoughts, feelings, attitudes, and behaviours of other people affect people living with dementia.<br>• To understand that the consequences of stigma and discrimination that people living with dementia experience are as important as knowing the disease itself.<br>• To understand the power of language as a mechanism of prejudice and discrimination.<br>• To identify possible behaviours and inadequate attitudes towards dementia and people living with dementia among participants.	• The importance of language, what is said and what is not said, in relation to people living with dementia, as well as common stigma and discrimination practices.<br>• Commonly used terms that reproduce stigma.	• This will be a dynamic session in which we ask questions to the group in one slide, and provide feedback in the following slide, and so on.<br>• Re-read and hold a debate on the answers participants gave on the first day, prior to the intervention start and compare them with the views they have now about people living with dementia and their carers.

For peer review only - http://bmjopen.bmj.com/site/about/guidelines.xhtml

	Session 2	• To understand that people living with dementia have desires, preferences, feelings, and aspirations.<br>• To sensitize participants about common negative behaviours, thoughts, and attitudes towards people living with dementia.<br>• To develop empathy for the everyday issues experienced by people living with dementia.	• The heterogeneity of people living with dementia.<br>• Importance of looking at people who live with dementia in a holistic way, beyond the disease.<br>• Compassion, understanding, and empathy for everyday situations experienced by people living with dementia.	• Show video narratives of people living with dementia<br>• Hand out two case vignettes to participants in which we depict two cases of people who develop dementia, and they have to read these in 'first person' as if the story described were theirs<br>• Hold a group discussion about how they felt in reading those experiences and about how they would have liked to be treated.
**Day 3:** Developing compassion and empathy and strengthening communication and behavioural skills.	Session 1	• To explain and demonstrate positive verbal and non-verbal communication strategies.<br>• To apply these strategies in groups.<br>• To actively try to recognise possible transformations in their beliefs, attitudes, and behaviours related to people living with dementia compared to the beginning of the intervention.	• Practical strategies for reflection on appropriate ways of caring for people living with dementia.<br>• Positive verbal and non-verbal communication strategies.	• Content display using slides.
	Session 2	• To identify inappropriate behaviours and attitudes in their own practices.<br>• To reflect on how to apply positive verbal and non-verbal communication strategies learned in the previous session.<br>• To generate feedback on the six sessions held.	• Importance of the CHWs work in combating the stigma of dementia.	• Real-life-based stories about the daily life of people living with dementia are presented and discussed.<br>• Reflections and discussion of the theoretical contents discussed in the three days of intervention.

For peer review only

The group intervention will seek to improve knowledge about dementia, as well as attitudes and behaviours towards people living with dementia and their carers. Table 2 presents the theory of change to be tested in this intervention, including "causes of problems", "problems", "resources", "activities or actions", "mechanisms of change", and "expected outcomes". This logic model was developed based on the literature related to stigma and dementia, as well as on exploratory work conducted previously in Brazil including experts by experience.[9,26] This has been presented and discussed among the international and multidisciplinary (*research programme name*) team for internal validation.[32]

For peer review only

For peer review only - http://bmjopen.bmj.com/site/about/guidelines.xhtml

Table 2. Theory of change: stigma and discrimination related to dementia among community health workers

**General parameters**	**Type of program**<br>Feasibility study	**Modality**<br>Psychoeducational	**Target population**<br>Community health workers	**Mode**<br>Group based	**Local**<br>Primary healthcare units	**City**<br>São José dos Campos, São Paulo

**Problems**	**Poor knowledge and beliefs**<br>○ Dementia is a natural part of aging<br>○ Dementia only affects older people<br>○ Every person who lives with dementia was born with a predisposition to dementia<br>○ The person is to blame for the development of dementia (e.g. lifestyle)<br>○ The brain has not developed<br>○ Nothing can be done to support people living with dementia and their carers	**Stereotypes and labelling**<br><u>Visions that people living with dementia are:</u><br>○ Dependent and incapable<br>○ Passive<br>○ Depressed and fragile<br>○ Isolated and alone<br>○ Crazy and unpredictable<br>○ Aggressive<br>○ Difficult to deal with<br>○ Burdensome<br>○ Repetitive<br>○ Abnormal<br>○ Plaintiffs<br>○ Manipulative<br>○ Unable to have feelings	**Separation, blame, avoidance, loss of status, social exclusion**<br>○ The person is not allowed to make decisions and to engage in social\alone activities and is discouraged from maintaining social bonds and roles (e.g. work)<br>○ The person is no longer considered to be part of groups to which they were before dementia<br>○ The person has the value of their opinion and contribution reduced, and is ignored or avoided<br>○ The person is expected to obey and 'behave' and has their freedom removed and\or restricted<br>○ The person is blamed for the stigma and discrimination (e.g. excluded due to being considered repetitive)<br>○ The person suffers physical and emotional abuse<br>○ There is a reduction in social ties and participation<br>○ Carers feel isolated and excluded<br>○ Carers tend to keep the person living with dementia indoors to "protect" them	**Internalized stigma**<br>○ Low self-esteem<br>○ Doubt own feelings and experiences<br>○ Denial of dementia<br>○ Diagnosis concealment<br>○ Social withdrawn<br>○ Loss of identity<br>○ Reduced wellbeing	**Less access to health services**<br>**Poor quality services**<br>○ Lack of support services<br>○ Lack of training and qualified professionals<br>○ Reduced help seeking<br>○ Late diagnosis<br>○ Care mostly focused on physical needs<br>○ The care provided generates dependence and is passive<br>○ Care does not involve the visions and desires of the person living with dementia and their carers<br>○ Carers have high emotional and physical burden

**Intervention resources**	Audio-visual presentations	Video-narratives of people living with dementia and caregivers	Group discussions and reflections	Dosage: Six sessions, divided into three sequential meetings, lasting three hours per day (9h in all)

**Activities**	What is dementia and how does it affect people living with dementia and their carers?	Experiences of people living with dementia and their carers.	Stigma, stereotypes, attributes, and language: discriminatory practices and their consequences.	Human rights-based practices, person-centred, and ethical care.	Strengthening tangible skills of compassion, empathy, and communication (verbal and nonverbal).	How can I improve my CHWs practice to better serve people living with dementia and their carers?<br>Brief and general evaluation of the programme

**Outcomes 30 days after intervention**	Improvement of knowledge and attitudes	Improved attitudes towards people living with dementia and their caregivers	Development of person-centred skills	Reduction of stigma levels	Reduction of discrimination levels

Active intervention and control

The intervention will be undertaken in different sessions with the participants of each PCU allocated to the experimental group. The intervention will be led by DO and will consist of three group meetings, held on three consecutive days, lasting three hours each, involving all participating CHWs from each PCU in each meeting (total = 9 hours over three days for all CHWs) (Figure 1). The control group will not receive any activity and will continue with their usual routine. We will deliver the same activity after the end of the study to individuals from the control group who wish to receive it.

Assessment

Primary and secondary outcomes

The primary outcome will be the feasibility of the intervention, including the findings of a nested qualitative component. The secondary outcomes will be the stigma-related measures: knowledge, attitudes and intended behaviour. All the outcome measures are described as follows.

Baseline and post study assessment

Baseline (t0) and post study assessments (t2) will be the same for both experimental and control groups. Additional feasibility questions (closed and open questions) will be applied to the experimental group on the last day of the intervention as well as in t2, and 15 to 20 participants in the experimental group will be invited to participate in one-to-one semi-structured interviews. Apart from the qualitative interviews, the written questions are all self-administered and will be completed on paper. Questionnaires will be distributed and collected by an assistant researcher who will be blinded to participant allocation. The researchers applying the intervention will not have access to the participants' responses until the end of data collection. It is expected that CHWs will take 10 to 15 minutes to complete all the questions. Considering the acceptance of approximately 14 PCUs to participate in the study, it is estimated that the total period between t0 and t2 in all PCUs will be 90 days.

Sociodemographic and stigma-related outcomes

Sociodemographic questions will include age, gender, level of education, religion, as well as information on previous training and experience with dementia (details in Supplementary material I). Stigma-related questions (dementia knowledge, attitudes and intended behaviour towards people living with dementia) were derived from the 2019 Global Stigma and Dementia Survey (WAR). The WAR questionnaire has been administered to more than 70,000 people worldwide including healthcare professionals, members of the public, people living with dementia, and their carers, through which process it has been translated to

For peer review only - http://bmjopen.bmj.com/site/about/guidelines.xhtml

Brazilian Portuguese.[2] The WAR questions were designed in Likert scale format and are concerned with the dementia knowledge, attitudes and anticipated behaviour towards people living with dementia. The questions were informed by existing validated stigma and discrimination scales[33,34] and have been validated into a reduced number of items with high psychometric performance[2,35].

Feasibility assessment

First, a brief evaluation will be conducted on the last day of the intervention through discussion and notes will be taken by the researchers (e.g. relevance, positive and negative aspects). Then, in t2, additional measures (objective and subjective) regarding the feasibility of the intervention will be applied, including a measure of satisfaction with the intervention[36] and open questions (details in Supplementary material I). We will also collate information on the number of sessions carried out according to protocol, total number of participants and per session, participant characteristics, recruitment rate, intervention completion rate, evaluation measures completion, appropriateness/acceptability, and fidelity of the intervention.

Nested qualitative component

Two participants from each PCU participating in the experimental group (n~14), including those who dropped out the study, will be invited to voluntarily participate in individual semi-structured interviews to explore further aspects of feasibility and potential impact of the intervention (details in Supplementary material II). Interviews will take place on the same day of t2, after all the post-study measures have been completed. The sample will be selected purposively, aiming to include a variety of sociodemographic characteristics, locations, and background training on dementia. To reduce the potential selection bias based on the interpersonal experience of the researchers applying the intervention with the participants, another member of the team will make this selection based on the characteristics reported in the questionnaires, and another researcher will conduct the interview itself. The interviews will last a maximum of 60 min, will be voice recorded, and will be held in a private room at the PCUs where the CHWs work.

Data analysis

We will seek to adhere to the steps proposed in the modelling phase of the Medical Research Council guidelines for developing complex interventions.[37] The purpose of a feasibility study is not to make a formal analysis of the primary outcome, but to evaluate trial processes to determine whether to progress to a study of effectiveness and to estimate parameters needed to design the future trial.[37] Data analysis will be conducted by a

For peer review only - http://bmjopen.bmj.com/site/about/guidelines.xhtml

researcher who has not been involved with the intervention itself and who is blinded to the arm allocation. Double data entry and checking will be used to ensure accuracy.

We hypothesize that the intervention is feasible and might be able to improve knowledge, attitudes and intended behaviour in relation to dementia among CHWs. Analysis of quantitative data will be based on “intention to treat”, that is, data from all participants will be included regardless of their withdrawal from the study or not. Descriptive analysis will include central and dispersion measures, according to the types (e.g. continuous, categorical, nominal) and distribution patterns of the variables, including number of incomplete responses and dropouts, ceiling, and floor effects. Differences in levels of dementia knowledge, as well as attitudes and intended behaviours towards people living with dementia before and after the intervention between groups and between time points (t0 and t2) will be measured using Student's t test or a non-parametric test, according to data distribution. We will also measure internal reliability (Cronbach's alpha) and test-retest, levels of variance, correlation between sociodemographic variables, and between the different outcome variables.

Quantitative feasibility measures will be analysed descriptively and will be compared with the results from the outcome measures. Feasibility data collected through qualitative methods will be analysed in an integrated manner using content analysis as well as triangulation techniques in NVivo®.[38,39] In preparation to that, semi-structured interviews will first be transcribed anonymously and verbatim.

Ethic statement

This study was approved by the National Commission for Ethics in Research in Brazil (CONEP) (*number removed for peer review*). Every individual taking part will be informed about their rights as participants, including the fact that nonparticipation will not affect in any way their work status or care received at the PCU. Every participant will sign a consent form; those participating in the semi structured interviews will be required to sign a second consent form specific for this research activity. Safety monitoring procedures have been created to protect participants and researchers, including safety measures to prevent emotional impact and the spread of COVID-19 (e.g. mandatory vaccination and use of mask during any study activity, as well as physical distance during the study).

Patient and public involvement

Through a series of focus groups and semi-structured interviews, we have explored the views of family carers, people living with dementia, health care workers and members of the

For peer review only - http://bmjopen.bmj.com/site/about/guidelines.xhtml

public about stigma and discrimination related to people living with dementia. The findings from this exploratory activity have informed the development of the intervention. We have also presented and discussed this intervention protocol among the international and multidisciplinary (*research programme name*) team for internal validation.[32] As this is a protocol for a feasibility study which involves both quantitative and quantitative procedures, we aim to gather participants' views about the intervention as part of the study itself in order to improve it for a future trial. We will also continue to consult our international consortium, which includes experts by experience, about the various methodological and practical aspects of the work as it progresses.

Dissemination plan

We plan to disseminate this study in open access scientific and community events related to dementia. The intervention materials will be published online and will be available for use by anyone who wishes to translate, adapt, and implement it. Our research team works closely with policy makers as part of a national advisory team, which we hope will help increase the possibility of such intervention to be applied in more settings in Brazil. An anti-stigma intervention toolkit will be informed by this study will be accessible nationally in Brazil and globally through open access publication to support researchers and practitioners with the implementation of dementia-related anti-stigma actions.

Discussion

The number of people living with dementia in Brazil is likely to increase in the next decades, however the number of people who are undiagnosed remains high and there is limited access and availability of appropriate support. Improving knowledge, attitudes, and behaviour of healthcare workers towards people living with dementia is a global health priority. In doing so, this is likely to reduce the stigma and discrimination experienced by people living with the condition, protect the quality of their lives, improve diagnosis rates, and quality care. Anti-stigma interventions have been successful in other countries and disease contexts, such as other mental health conditions and HIV. However, there is a paucity of research on anti-stigma interventions related to dementia, particularly targeting health care workers.

To our knowledge, this is the first anti-stigma intervention related to dementia to be conducted in Brazil and we are not aware of any other similar intervention in other Latin American countries. We will target CHWs who are the first point of healthcare access for people living with dementia in the community and we hope this will help increase the number of people living with dementia who are identified and attended by the healthcare systems. As

For peer review only

CHWs work alongside multidisciplinary teams, we hope that the learning experiences achieved through this intervention will be shared among other members of the team, contributing to a further reach of the intervention and higher impact. The measures of feasibility included in this protocol will be paramount to ensure that the invention is acceptable, relevant, and effective to be applied in a future randomized controlled trial in Brazil and in other LMICs. An anti-stigma toolkit will be created from the intervention will also help ensure fidelity of future trials, as well as applicability in PCUs whose teams have the opportunity to apply it in their territories.

Author contribution: DO has led the design and development of the intervention, and the writing of the manuscript; CG has supported with the development of the intervention and has contributed to the writing of the manuscript; FAFM, EM, ACF, NF, SEL, and CPF have supported with the design and development of the intervention and have contributed to the writing of the manuscript.

Abbreviations

CHWs: Community Health Workers

CONEP: National Commission for Ethics in Research

LMICs: Low- and Middle-Income Countries

PCU: Primary Healthcare Unit

Availability of data and materials

Reasonable requests for the datasets used and/or analysed during the current study will be considered by the study team.

Funding

This project is supported by UK Research and Innovation Global Challenges Research Fund (ES/P010938/1).

Competing interests

The authors declare no conflict of interest.

References

1. World Health Organization. Global status report on the public health response to dementia [Internet]. Geneva: World Health Organization; 2021 [cited 2021 Sep 9]. Available from: https://apps.who.int/iris/handle/10665/344701

For peer review only - http://bmjopen.bmj.com/site/about/guidelines.xhtml

2. Alzheimer Disease International. World Alzheimer Report 2019: Attitudes to dementia [Internet]. 2019 [cited 2020 May 11]. Available from: https://www.alz.co.uk/research/world-report-2019

3. Alzheimer Disease International. World Alzheimer Report 2021: Journey through the diagnosis of dementia [Internet]. 2021. Available from: (in press)

4. Link BG, Phelan J. Stigma power. Social Science & Medicine. 2014 Feb;103:24–32.

5. Zimmermann M. Alzheimer's Disease Metaphors as Mirror and Lens to the Stigma of Dementia. Lit Med. 2017;35(1):71–97.

6. Nguyen T, Li X. Understanding public-stigma and self-stigma in the context of dementia: A systematic review of the global literature. Dementia. 2020 Feb;19(2):148–81.

7. Rüsch N, Angermeyer MC, Corrigan PW. Mental illness stigma: Concepts, consequences, and initiatives to reduce stigma. Eur psychiatr. 2005 Dec;20(8):529–39.

8. Werner P. Stigma and Alzheimer's disease: A systematic review of evidence, theory, and methods. The stigma of disease and disability: Understanding causes and overcoming injustices. 2014;223–44.

9. Oliveira D, Da Mata FAF, Mateus E, Musyimi CW, Farina N, Ferri CP, et al. Experiences of stigma and discrimination among people living with dementia and family carers in Brazil: qualitative study. Ageing and Society. 2021 May 20;1–22.

10. Evans SC. Ageism and Dementia. In: Ayalon L, Tesch-Römer C, editors. Contemporary Perspectives on Ageism [Internet]. Cham: Springer International Publishing; 2018. p. 263–75. Available from: https://doi.org/10.1007/978-3-319-73820-8_16

11. Thornicroft G, Mehta N, Clement S, Evans-Lacko S, Doherty M, Rose D, et al. Evidence for effective interventions to reduce mental-health-related stigma and discrimination. The Lancet. 2016 Mar 12;387(10023):1123–32.

12. Thornicroft G, Brohan E, Rose D, Sartorius N, Leese M, INDIGO Study Group. Global pattern of experienced and anticipated discrimination against people with schizophrenia: a cross-sectional survey. Lancet. 2009 Jan 31;373(9661):408–15.

13. Semrau M, Evans-Lacko S, Koschorke M, Ashenafi L, Thornicroft G. Stigma and discrimination related to mental illness in low- and middle-income countries. Epidemiol Psychiatr Sci. 2015 Oct;24(5):382–94.

14. Lopez RP, Rose KM, Kenney L, Sanborn V, Davis JD. Managing Shame: A Grounded Theory of How Stigma Manifests in Families Living With Dementia. J Am Psychiatr Nurses Assoc. 2019;(9507418):1078390319832965.

15. Schmutte T, Olfson M, Maust DT, Xie M, Marcus SC. Suicide risk in first year after dementia diagnosis in older adults. Alzheimer's & Dementia. 2021 May 25;alz.12390.

16. Brossard B. Losing credibility. In: Forgetting Items: The Social Experience of Alzheimer's Disease [Internet]. Indiana University Press; 2017 [cited 2020 Aug 4]. p. 35–70. Available from: https://www.jstor.org/stable/j.ctvpb3z16.6

For peer review only

17. Hatzenbuehler ML. Structural stigma: Research evidence and implications for psychological science. American Psychologist. 2016 Nov;71(8):742–51.

18. Clay J, Eaton J, Gronholm PC, Semrau M, Votruba N. Core components of mental health stigma reduction interventions in low- and middle-income countries: a systematic review. Epidemiol Psychiatr Sci. 2020 Sep 4;29:e164.

19. Prins M, Veerbeek M, Willemse BM, Pot AM. Use and impact of the Alzheimer Experience: a free online media production to raise public awareness and enhance knowledge and understanding of dementia. Aging & Mental Health. 2019 Mar 5;1–8.

20. Farina N, Suemoto CK, Burton JK, Oliveira D, Frost R. Perceptions of dementia amongst the general public across Latin America: a systematic review. Aging & Mental Health. 2020 Feb 12;1–10.

21. Ibanez A, Flichtentrei D, Hesse E, Dottori M, Tomio A, Slachevsky A, et al. The power of knowledge about dementia in Latin America across health professionals working on aging. Alzheimer's & Dementia: Diagnosis, Assessment & Disease Monitoring [Internet]. 2020 Jan [cited 2020 Oct 20];12(1). Available from: https://onlinelibrary.wiley.com/doi/10.1002/dad2.12117

22. Nakamura AE, Opaleye D, Tani G, Ferri CP. Dementia underdiagnosis in Brazil. The Lancet. 2015 Jan;385(9966):418–9.

23. Fagundes SD, Silva MT, Thees MFRS, Pereira MG. Prevalence of dementia among elderly Brazilians: a systematic review. Sao Paulo Med J. 2011 Jan 6;129(1):46–50.

24. OPAS/PAHO. Mental Health [Internet]. 2020 [cited 2021 Jan 27]. Available from: https://www.paho.org/en/topics/mental-health

25. Patel V, Maj M, Flisher AJ, De Silva MJ, Koschorke M, Prince M, et al. Reducing the treatment gap for mental disorders: a WPA survey. World Psychiatry. 2010 Oct;9(3):169–76.

26. Oliveira D, et al. Stigma and discrimination related to people living with dementia among healthcare workers and members of the public in Brazil: Qualitative study. in preparation. n\d;

27. World Health Organization. WHO QualityRights Tool Kit [Internet]. 2012 [cited 2021 Feb 4]. Available from: https://www.who.int/mental_health/publications/QualityRights_toolkit/en/

28. Brooker DJ, Latham I. Person-Centred dementia care: Making services better with the VIPS Framework. 2nd ed. London: Jessica Kingsley Publishers; 2016.

29. Edwards R, Voss S, Iliffe S. Education about dementia in primary care: is person-centredness the key?. DEMENTIA. 2014;13(1):111–9.

30. Kitwood T. Malignant social psychology. In: Understanding care, welfare and community a reader [Internet]. London; New York: Routledge; 2006 [cited 2021 Feb 3]. Available from: https://ebookcentral.proquest.com/lib/ulaval/detail.action?milDocID=11111

31. North J. Achieving Person-Centred Health Systems: Evidence, Strategies and Challenges [Internet]. 1st ed. Nolte E, Merkur S, Anell A, editors. Cambridge University

For peer review only - http://bmjopen.bmj.com/site/about/guidelines.xhtml

Press; 2020 [cited 2020 Dec 28]. Available from: https://www.cambridge.org/core/product/identifier/9781108855464/type/book

32. Ghate D. Developing theories of change for social programmes: co-producing evidence-supported quality improvement. Palgrave Commun. 2018 Dec;4(1):90.

33. Bakolis I, Thornicroft G, Vitoratou S, Rüsch N, Bonetto C, Lasalvia A, et al. Development and validation of the DISCUS scale: A reliable short measure for assessing experienced discrimination in people with mental health problems on a global level. Schizophrenia Research. 2019 Oct;212:213–20.

34. Brohan E, Clement S, Rose D, Sartorius N, Slade M, Thornicroft G. Development and psychometric evaluation of the Discrimination and Stigma Scale (DISC). Psychiatry Research. 2013 Jun;208(1):33–40.

35. D'Amico F, Bhatt J, Comas-Herrera A, Farina N, Gaber S, Knapp M, et al. Development and validation of a knowledge scale for stigma in dementia using responses from the general public. (submmited for publication). 2022;

36. Wang Y, Xiao LD, Ullah S, He G-P, De Bellis A. Evaluation of a nurse-led dementia education and knowledge translation programme in primary care: A cluster randomized controlled trial. Nurse Education Today. 2017 Feb;49:1–7.

37. O'Cathain A, Croot L, Duncan E, Rousseau N, Sworn K, Turner KM, et al. Guidance on how to develop complex interventions to improve health and healthcare. BMJ Open. 2019 Aug;9(8):e029954.

38. Bengtsson M. How to plan and perform a qualitative study using content analysis. NursingPlus Open. 2016;2:8–14.

39. Erlingsson C, Brysiewicz P. A hands-on guide to doing content analysis. African Journal of Emergency Medicine. 2017 Sep 1;7(3):93–9.

For peer review only

Figure 2. Study flow

For peer review only

For peer review only - http://bmjopen.bmj.com/site/about/guidelines.xhtml

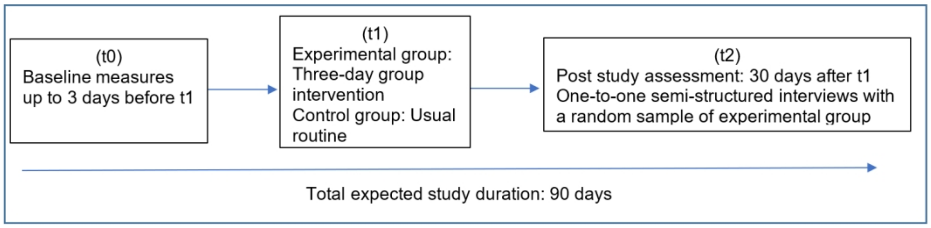

Figure 1. Study flow

Figure 1. Study flow

757x217mm (57 x 57 DPI)

Supplementary material I

Baseline and post-study assessment

Sociodemographic and work-related data

1. Date of birth: _____________
2. Age: _______
3. Sex: Female ( ) Male ( ) Prefer not to say ( )
4. School attendance:
   Primary school ( ) Middle school ( ) Undergraduate degree or above ( )
5. Religion: None ( ) Catholic ( ) Evangelical ( ) Other: _________________
   Prefer not to say ( )
6. Race: Black ( ) White ( ) Mixed-race ( ) Yellow ( ) Prefer not to say ( )
7. PCU name: ____
8. Number of months\years working as a CHW ___________________________
9. Do you have any <u>personal</u> experience working with people living with dementia or carers?
   Yes ( ) No ( ) Does not know or cannot remember ( )
10. Do you have any <u>professional</u> experience working with people living with dementia or carers?
    Yes ( ) No ( ) Does not know ( ) Cannot remember ( )
11. Have you ever had any training on dementia?
    Yes ( ) No ( ) Does not know ( ) Cannot remember ( )
12. If you said yes to the question above, when was this training and what was the focus?
    Month and year: __________________________
    Topic: ____

Global Survey on Stigma and Dementia (2019)

Questions on knowledge, attitudes and intended behaviours related to people living with dementia. Items available upon request: https://www.alzint.org/resource/world-alzheimer-report-2019/

Feasibility

For peer review only

Satisfaction with the intervention (Wang et al., 2017)

Instruction: Please read each of the items below and circle the answer that best reflects your opinion. We are trying to improve the intervention you participated in, so please be as honest as possible with your answers so we can do better in the future.

Questions	Strongly disagree	Disagree	I neither agree nor disagree	Agree	Strongly agree
1. Before participating in the program, my knowledge about dementia and caring for people living dementia was very limited.	1	2	3	4	5
2. This program has helped me learn new knowledge on dementia and care for people living with dementia.	1	2	3	4	5
3. The activities were necessary and useful.	1	2	3	4	5
4. The discussions improved my learning in relation to people living with dementia.	1	2	3	4	5
5. Discussions on how to translate knowledge into practice helped me think about how to apply knowledge in my practice.	1	2	3	4	5
6. The materials were clear and informative.	1	2	3	4	5
7. Overall, the program has reached my expectations.	1	2	3	4	5
8. Overall, I believe the program will help improve my work with people living with dementia and their carers.	1	2	3	4	5

For peer review only

Open questions

Please answer the questions below with your opinions about your participation in this project. We are trying to improve the intervention you participated in, so please be as honest as possible with your answers so we can do better in the future.

1. Would you recommend this program for another CHW? Why?

2. Overall, from 0 to 10, how satisfied were you with your participation in the program? Why? (0- no satisfied at all, 10- very satisfied)

3. What did you like the most in this programme? Why?

4. What did you not like in this programme? Why?

5. Was there anything that got in the way of your participation? If so, what was it?

6. What\How could the program be improved?

7. Have you talked about any of the aspects discussed in this programme with anyone?

8. How do you think participation in this program may have affected or will affect your practice as CHW?

9. Could you give a concrete example of any change that has already occurred in your CHW's activities in relation to people living with dementia and their families after your participation in the program? If you believe there has been no change, answer "no."

For peer review only

Supplementary material II

Interview schedule

Introduction

1. Thank the participant for taking part

2. The purpose of the interview

I'm part of a project called STRiDE. The idea of the project is …

We would like to hear your ideas and suggestions related to your participation in this project, so I'm going to ask you a few questions about this. I have here a list of questions and topics that will serve as a general guide on what I would like to talk to you about today, but this is flexible since the most important thing is that we talk about what is important to you.

The duration of the interview depends on your availability and interest. It usually lasts 60 min, but we can use more or less time. You can stop the interview at any time if you don't want to continue. It's okay if you want to take a break when you feel the need.

3. Reminder of ethical issues related to consent, anonymity, confidentiality, and safety measures

4. Presentations

5. Questions

Is there anything else you'd like to know about your participation or any other questions you may have?

6. Interview

1. Why did you decide to participate in this study?
2. What do you remember the most of what we covered in the sessions?
3. How would you describe the training and key messages covered?
4. Could tell me about any learning points you had through this programme?
5. What did you like the most and what did you like the least about this program?
6. Do you think the duration of the sessions and the whole study were sufficient or should be shorter or longer? Why is that?
7. If you could participate again, what would you like to be different?
8. What do you think we could do to get as many CHWs as possible involved in activities like this?
9. Do you consider that your participation in the programme is likely to change your practice as a CHW with people living with dementia and their carers in any way? Why\How?
10. Do you think participating in this programme and applying what you've learned in practice can help improve the care provided for people living with dementia and their carers in any way in real life? Why\How?
11. In your opinion, is there anything you think would limit how much you can put in practice what you learned during the intervention? Could you explain why?

For peer review only

12. In relation to the previous question, how could we researchers mitigate such factors?

13. Would you recommend this program to someone? Why is that?

14. Do you have any other comments to make about the intervention?

End of interview

1. Remind the participant about the end of the interview.
2. Thank the participant for their valuable contributions.
3. Tell the participant what will happen now (e.g. how the data will be used).

For peer review only

BMJ Open

Reducing dementia-related stigma and discrimination amongst community health workers in Brazil: Protocol for a randomized controlled feasibility trial

Journal:	*BMJ Open*
Manuscript ID	bmjopen-2021-060033.R1
Article Type:	Protocol
Date Submitted by the Author:	09-May-2022
Complete List of Authors:	Oliveira, Deborah; Universidade Federal de São Paulo, School of Medicine, Department of Psychiatry<br>Godoy, Carolina; Universidade Federal de São Paulo, School of Medicine, Department of Psychiatry<br>da Mata, Fabiana ; Universidade Federal de São Paulo, School of Medicine, Department of Psychiatry<br>Mateus, Elaine; Brazilian Federation of Alzheimer's Associations; Universidade Estadual de Londrina (UEL), Applied Linguistics<br>Franzon, Ana Carolina; Brazilian Federation of Alzheimer's Associations<br>Farina, Nicolas; Brighton and Sussex Medical School, Centre for Dementia Studies<br>Evans-Lacko, Sara; London School of Economics and Political Science, Personal Social Services Research Unit<br>Ferri, Cleusa; Universidade Federal de Sao Paulo, School of Medicine, Department of Psychiatry; Hospital Alemão Oswaldo Cruz, Health Technology Assessment Unit
**Primary Subject Heading**:	General practice / Family practice
Secondary Subject Heading:	Mental health, Global health, Geriatric medicine
Keywords:	Dementia < NEUROLOGY, GERIATRIC MEDICINE, MENTAL HEALTH, PRIMARY CARE, Delirium & cognitive disorders < PSYCHIATRY, Old age psychiatry < PSYCHIATRY

SCHOLARONE™
Manuscripts

Reducing dementia-related stigma and discrimination amongst community health workers in Brazil: Protocol for a randomized controlled feasibility trial

Authors: Déborah Oliveira[1], Carolina Godoy[1], Fabiana A. F. da Mata[1], Elaine Mateus[2,3], Ana Carolina Arruda Franzon[2], Nicolas Farina[4], Sara Evans-Lacko[5], Cleusa P. Ferri[1,6]

Affiliations:

1. Universidade Federal de São Paulo (UNIFESP), School of Medicine, Department of Psychiatry. São Paulo, Brazil.
2. Brazilian Federation of Alzheimer's Associations (FEBRAZ). Paraná, Brazil.
3. Universidade Estadual de Londrina (UEL), Applied Linguistics, Department of Modern Languages, Paraná, Brazil.
4. Brighton and Sussex Medical School, Centre for Dementia Studies, Brighton, United Kingdom.
5. London School of Economics and Political Science, Care Policy and Evaluation Centre, London, United Kingdom.
6. Hospital Alemão Oswaldo Cruz, Health Technology Assessment Unit, São Paulo, Brazil.

Corresponding author: Déborah Oliveira. Universidade Federal de São Paulo (UNIFESP), School of Medicine, Department of Psychiatry. Rua Major Maragliano, 241, Vila Mariana, Post Code 04017-030, São Paulo, Brazil. E-mail: oliveiradc.phd@gmail.com

Word count: 3,968

Abstract

Introduction: Stigma and discrimination amongst healthcare workers can hinder diagnosis and the provision of appropriate care in dementia. This study is aimed at developing, delivering, and evaluating the feasibility of a group anti-stigma intervention to improve knowledge, attitudes, and behaviours in relation to people living with dementia among Community Health Workers (CHWs). **Methods and analysis:** This will be a randomized controlled feasibility trial conducted with 150 CHWs from 14 Primary Care Units (PCUs) in São Paulo, Brazil. PCUs will be randomly allocated (1:1) in two parallel groups - experimental group or control group. Participants from PCUs allocated to the experimental group will receive a three-day group intervention involving audio-visual and printed materials, as well as elements of social contact. The control group will keep their usual routine. Knowledge, attitude and intended behaviour stigma-based outcomes will be assessed at baseline and at follow up (30 days after intervention) to both groups, with additional

For peer review only - http://bmjopen.bmj.com/site/about/guidelines.xhtml

questions on feasibility for the experimental group at follow up. Around 10-15 participants will take part in follow up semi-structured interviews to further explore feasibility. Quantitative analyses will follow an “intention to treat” approach. Qualitative data will be analysed using content analysis. **Ethics and dissemination:** This study was approved by the National Commission for Ethics in Research in Brazil (*number*). Every participant will sign a consent form. Results will be disseminated through academic journals and events related to dementia. The intervention materials will be made available online.

Keywords: dementia; stigma; feasibility studies; primary care; community health workers

For peer review only

Strengths and limitations of this study

- This is the first study to develop, deliver and evaluate a controlled feasibility study of a group anti-stigma intervention to improve knowledge, attitudes, and behaviours in relation to people living with dementia among community health workers in Brazil.
- This study will seek to involve all the community health workers of all the primary care units of a large urban city in São Paulo, Brazil.
- The study includes quantitative and qualitative components that will assess the feasibility of the intervention from different perspectives and will include a follow up assessment which will help us understand any potential long-term impact.
- As this will be a feasibility study, no conclusions can be drawn about the effectiveness of the intervention, however we hope the information collected will help build a robust future randomized controlled trial.

Background

There are approximately 55.2 million people living with dementia worldwide and nearly 70% of this population live in low- and middle-income countries (LMICs), such as Brazil.[1] By 2050, this number is expected to increase to 139 million.[1] Stigma and discrimination related to dementia are common, which is particularly important among healthcare workers, considering the detrimental impact on the lives of those living with the condition and their families, such as by hindering access to diagnosis and support.[2,3] Combating the stigma related to dementia is a global health priority.[1]

Stigma occurs when a label associated with a negative stereotype is attributed to an individual characteristic, causing people with such characteristics to be seen as separate and of lower status compared to people without the characteristic.[4] When stigma occurs, 'power' is exercised by stigmatisers to keep stigmatized groups 'down' or dominated/

For peer review only - http://bmjopen.bmj.com/site/about/guidelines.xhtml

exploited; 'within', to maintain social norms; and 'away' by means of social exclusion.[5] In dementia, stigmatization occurs through negative stereotypes, related to cognitive decline can lead to depersonalization and considering the person as unable to continue to live in and contribute to society.[6–9] Although not every person living with dementia is an older person, older people living with dementia are likely to experience stigma related to dementia, as well as from ageism and ableism, further impacting their rights and wellbeing.[10]

Several studies provide robust evidence on the negative impact that stigma and discrimination has on people living with mental disorders more broadly.[11–13] Although there is a paucity of research on the impact of stigma on people living with dementia,[6] the existing evidence shows that stigma can lead to negative feelings about one self, shame, symptom and diagnosis concealment, negative social interactions, reduced access to care networks and social participation, and even suicide. [2,6,9,14–16] A global survey on attitudes towards dementia among members of the public, people living with dementia, family carers and healthcare professionals from 155 countries showed that over 85% of respondents living with dementia had their opinion not taken seriously, as well as between 35% and 57% (in high-income countries and LMICs, respectively) had been treated unfairly in intimate relationships. Moreover 40% of the general public believed health professionals ignore people living with dementia; and 35% of carers reported hiding the diagnosis of dementia of a family member.[2]

Limited knowledge about dementia, as well as widespread misbeliefs (e.g. believing that dementia is a natural part of ageing) and negative attitudes (e.g. considering people living with dementia are burdensome) may make health professionals less likely to detect dementia and provide adequate care to people affected by this condition.[3,9] In over 75% of cases globally, diagnosis of dementia is either not made, or is made at a later stage, when the person living with the condition is no longer able to make decisions about their life and wellbeing independently.[3] Structural forms of stigma – such as when systems, policies, or services are designed in a way that discriminates directly or indirectly against people living with dementia[17] – also contributes to poor healthcare provision in dementia, by means of lack of investment in appropriate services and in training for health and social care providers, who continue to be unprepared to diagnose and to provide care and support for people living with dementia,[3] further contributing to the experiences of stigma and discrimination experienced by these individuals. The current state of healthcare systems and providers can therefore be seen as a consequence, as well as a source of, stigma related to dementia.

For peer review only - http://bmjopen.bmj.com/site/about/guidelines.xhtml

A recent systematic review with 56 studies showed that most interventions to reduce mental health related stigma in LMICs are effective.[18] These were focused on stigma related to schizophrenia, suicide, depression, child and adolescent mental health, bipolar disorder, anorexia nervosa and post-traumatic stress disorder among health care workers, students, or on more than one group. Around 75% found a significant positive effect for all main stigma outcomes and 25% found a small positive effect for some but not all stigma outcomes. Among the moderate or high-quality studies (n = 38), ten found a significant long-term positive effect on stigma outcomes.

The most common approach is education; however, a wide variety of methods – from creative arts-based approaches to those which emphasise empowering people with mental health conditions – can be successful, including social contact interventions.[18] Currently, very little is known about interventions which reduce stigma related to dementia[19] and research on knowledge and attitudes related to dementia in Latin American countries is scarce.[20,21] There is an urgent need for further research on dementia-related stigma in different contexts and cultures.[1,6]

It has been estimated that 77% of people living with dementia in Brazil are not diagnosed.[22] Prevalence estimates of people living with dementia vary from 5.1% to 19.0% among those aged 65 and over.[23] Brazil still lacks well-funded and well-equipped health systems to meet the needs of the growing population of people living with dementia and their families. The country has a universal health system in which Primary Care Units (PCUs) are the first point of access for people who experience any physical or mental health problem. In most regions, PCUs have Community Health Workers (CHWs) who contribute to health promotion and disease prevention activities.[24] Most CHWs live in the same communities where they work, being a valuable point of contact and an intersection between health services and their communities. Health systems with CHWs have the potential to help expand the delivery of mental health care and close the existing mental health gap in LMICs.[25] However, the training activities offered to CHWs in Brazil is focused on the control of communicable diseases, maternal and child health and non-communicable physical diseases, such as diabetes and hypertension, with very little training dedicated to dementia. Providing CHWs with adequate knowledge on dementia, as well as on common stigmatising and discriminatory practices, and positive attitudes and behaviours towards people living with dementia may help increase the number of people living with dementia identified by the PCU, as well as the quality of the health care and support provided to these individuals.

Aim and objectives

For peer review only - http://bmjopen.bmj.com/site/about/guidelines.xhtml

The overarching aim is to design an anti-stigma intervention to reduce stigma and discrimination towards people living with dementia among CHWs in the city of São José dos Campos, Sao Paulo, Brazil and to pilot the intervention using a feasibility randomized controlled trial. The specific objectives are:

1. To develop a group-based intervention to improve knowledge about dementia, as well as attitudes and behaviours towards people living with dementia; and

2. To test the intervention in a randomized controlled feasibility study and evaluate the acceptability and feasibility of performing such an intervention in a future randomized controlled trial.

Methods and analysis

Study design

A randomized controlled feasibility study will be conducted as part of a large multinational research programme (*name omitted for peer review*) which aims to contribute to the improvement of care systems, treatment, and support for people living with dementia and their families in Brazil and other LMICs. An exploratory qualitative study was conducted previously involving semi-structured interviews and focus groups with people living with dementia, family carers, healthcare workers, and members of the public in three large cities in the state of Sao Paulo.[9,26] The findings from the study helped inform the content and design of this intervention (Tables 1 and 2). This protocol (Version 1; Date: 4 May 2022) was prepared in line with the SPIRIT check list (Supplementary material I).[27,28] The study protocol will be registered in the Brazilian National Registry of Clinical Trials (REBEC: https://ensaiosclinicos.gov.br/), which will include all items from the World Health Organization Trial Registration Data Set (https://www.who.int/clinical-trials-registry-platform/network/who-data-set).

Participants and sampling

All CHWs in each of the 20 eligible PCUs located in São José dos Campos will be invited to take part. Individuals working as a CHWs for less than three months will be excluded. Each PCU has approximately from 8 to 12 CHWs, totalling 160 to 240 potential participants. A proportion (30%) of refusals to participate and dropouts are expected (e.g., change in work schedules), therefore we estimate to include approximately 14 PCUs involving around 150 participants in the trial.

Method of generating the allocation sequence

For peer review only - http://bmjopen.bmj.com/site/about/guidelines.xhtml

Randomization and allocation will be conducted by blinded member of the research team at the PCU level using computer-generated random numbers. The PCUs whose managers agree to participate will be randomly allocated in two parallel groups – experimental group or control group – so that all CHWs of a given PCU who voluntarily accept to take part can be allocated in the same group forming a cluster and reducing the potential for contamination (e.g., sharing knowledge learnt in the intervention between PCUs). The allocation will be made randomly at 1:1 so that there is the same number of PCUs in each group, offering as much as possible balanced participation among CHWs from PCUs located in the various geographical regions, proportional to the total number of PCUs of each region (20%: north, 20%: south, 20%: east, 20%: west, 20%: centre). The characteristics of participants (e.g., age, gender, time working as CHW) will be compared between groups and controlled for statistically if significant differences are observed.

Before informing the PCUs about the group to which their CHWs have been allocated, every CHW wishing to participate will read and sign a consent form. Recruitment and enrolment will be conducted by a separate member of the team, who will be blinded to the allocation. Then, all participants will be invited to complete the baseline measures (t1). The experimental group will be informed about the dates and timings of the intervention and the control group will be told that they will be allocated to a waiting list. After the completion of the final assessments from both experimental group and control group (t2), the intervention will be offered to the control group.

Development of the content of the intervention

Participants within the experimental group will receive a group intervention created by the researchers – DO (Nurse, Brazil), CG (Gerontologist, Brazil), CPF (Psychiatrist and Epidemiologist, Brazil), FAFM (Physiotherapist, Brazil), EM (Linguist, Brazil), ACAF (Journalist, Brazil), NF (Psychologist and dementia researcher, England), and SE-L (Stigma expert, England). The researchers have extensive experience in research and clinical practice with people living with dementia, CHWs and family carers. The intervention materials were informed by a rapid scoping review conducted by the team on dementia-related stigma in Latin America and the Caribbean (unpublished) and on the exploratory qualitative work conducted previously with people living with dementia, family carers, healthcare providers (including CHW), and members of the public in Brazil.[9,26] This was also supplemented with the literature on ethical and patient-centred care for people living with some type of mental and/or neuropsychiatric disorder,[29] literature in the area of person-centred care for people living with dementia,[30–33] as well as by evidence for effective interventions to reduce stigma in LMICs.[11,13,18]

For peer review only - http://bmjopen.bmj.com/site/about/guidelines.xhtml

The researchers will use audio-visual and printed materials containing, for example, videos of people with dementia and carers sharing their personal experiences, reflexive activities, group discussions, and presentations through Power Point® (Table 1).

For peer review only

For peer review only - http://bmjopen.bmj.com/site/about/guidelines.xhtml

Table 1. Summary of the intervention components and activities

When		Why: what we want to achieve	What: topic covered	How: method or strategy
**Day 1:** Building knowledge and demystifying dementia: beginning the transformation process.	Session 1	• To understand the general structure of the intervention and get to know one another, which will be important to build a non-judgmental space of trust among the individuals and for their spontaneous contribution to the activities.<br>• To get in touch with individual and shared beliefs and questions related to dementia, and to gain a better understanding of the condition and of the 'individual behind the disease'.	• What is dementia<br>• What is not dementia<br>• How people living with dementia are seen.	• Before the session starts, we ask participants to individually write down their views on dementia and people living with dementia using directed questions.<br>• We provide an interactive presentation on knowledge and common beliefs related to dementia.<br>• Each participant reads their views and shares with the group voluntarily. The group reflects and discusses these based on the presentation delivered earlier.
	Session 2	• To understand what dementia is, what dementia is not, and what treatment and care possibilities exist.<br>• To understand how dementia can affect people living with dementia and their families.<br>• To experience, through group dynamics, the impacts caused by prejudice, discrimination, and negative language on the lives of people living with dementia.	• What is dementia<br>• What is not dementia<br>• Known and unknown, modifiable, and non-modifiable risk factors.<br>• Pharmacological and non-pharmacological interventions can help people who live with dementia and their carers to have quality of life.<br>• Management and control of dementia symptoms.	• This will be a dynamic session in which we ask questions to the group in one slide, and provide feedback in the following slide, and so on.<br>• After, we will have the "secret box" dynamic: we will distribute individual boxes containing two photos of famous people and a mirror at the end. We ask participants to describe the characteristics of the two people and then to describe themselves. We try to identify differences in how they would describe 'others' and how they would describe 'themselves' to stimulate empathetic and non-judgemental approaches.<br>• We will hold a reflective and introductory debate on stigma, prejudice, discrimination, and use of language.<br>• We will promote reflection on how the participants felt when placing themselves in the 'shoes' of someone living with dementia.
**Day 2:** Breaking down labels and stereotypes and improving attitudes towards people living with dementia.	Session 1	• To understand how thoughts, feelings, attitudes, and behaviours of other people affect people living with dementia.<br>• To understand that the consequences of stigma and discrimination that people living with dementia experience are as important as knowing the disease itself.<br>• To understand the power of language as a mechanism of prejudice and discrimination.<br>• To identify possible behaviours and inadequate attitudes towards dementia and people living with dementia among participants.	• The importance of language, what is said and what is not said, in relation to people living with dementia, as well as common stigma and discrimination practices.<br>• Commonly used terms that reproduce stigma.	• This will be a dynamic session in which we ask questions to the group in one slide, and provide feedback in the following slide, and so on.<br>• Re-read and hold a debate on the answers participants gave on the first day, prior to the intervention start and compare them with the views they have now about people living with dementia and their carers.

For peer review only - http://bmjopen.bmj.com/site/about/guidelines.xhtml

	Session 2	• To understand that people living with dementia have desires, preferences, feelings, and aspirations.<br>• To sensitize participants about common negative behaviours, thoughts, and attitudes towards people living with dementia.<br>• To develop empathy for the everyday issues experienced by people living with dementia.	• The heterogeneity of people living with dementia.<br>• Importance of looking at people who live with dementia in a holistic way, beyond the disease.<br>• Compassion, understanding, and empathy for everyday situations experienced by people living with dementia.	• Show video narratives of people living with dementia<br>• Hand out two case vignettes to participants in which we depict two cases of people who develop dementia, and they have to read these in 'first person' as if the story described were theirs<br>• Hold a group discussion about how they felt in reading those experiences and about how they would have liked to be treated.
**Day 3:** Developing compassion and empathy and strengthening communication and behavioural skills.	Session 1	• To explain and demonstrate positive verbal and non-verbal communication strategies.<br>• To apply these strategies in groups.<br>• To actively try to recognise possible transformations in their beliefs, attitudes, and behaviours related to people living with dementia compared to the beginning of the intervention.	• Practical strategies for reflection on appropriate ways of caring for people living with dementia.<br>• Positive verbal and non-verbal communication strategies.	• Content display using slides.
	Session 2	• To identify inappropriate behaviours and attitudes in their own practices.<br>• To reflect on how to apply positive verbal and non-verbal communication strategies learned in the previous session.<br>• To generate feedback on the six sessions held.	• Importance of the CHWs work in combating the stigma of dementia.	• Real-life-based stories about the daily life of people living with dementia are presented and discussed.<br>• Reflections and discussion of the theoretical contents discussed in the three days of intervention.

For peer review only - http://bmjopen.bmj.com/site/about/guidelines.xhtml

The group intervention will seek to improve knowledge about dementia, as well as attitudes and behaviours towards people living with dementia and their carers. Table 2 presents the theory of change to be tested in this intervention, including "causes of problems", "problems", "resources", "activities or actions", "mechanisms of change", and "expected outcomes". This logic model was developed based on the literature related to stigma and dementia, as well as on exploratory work conducted previously in Brazil including experts by experience.[9,26] This has been presented and discussed among the international and multidisciplinary (*research programme name*) team for internal validation.[34]

Active intervention and control

The intervention will be undertaken in different sessions with the participants of each PCU allocated to the experimental group. The intervention will be led by DO and will consist of three group meetings, held on three consecutive days, lasting three hours each, involving all participating CHWs from each PCU in each meeting (total = 9 hours over three days for all CHWs) (Figure 1). This schedule was organized in a way that would improve participant's adherence to intervention as they will need to be off duty during the study sessions. The control group will not receive any activity and will continue with their usual routine. We will deliver the same activity after the end of the study to individuals from the control group who wish to receive it.

Assessment

Primary and secondary outcomes

The primary outcome will be the feasibility of the intervention, including the findings of a nested qualitative component. The secondary outcomes will be the stigma-related measures: knowledge, attitudes and intended behaviour. All the outcome measures are described as follows.

For peer review only - http://bmjopen.bmj.com/site/about/guidelines.xhtml

Table 2. Theory of change: stigma and discrimination related to dementia among community health workers

**General parameters**	**Type of program**<br>Feasibility study	**Modality**<br>Psychoeducational	**Target population**<br>Community health workers	**Mode**<br>Group based	**Local**<br>Primary healthcare units	**City**<br>São José dos Campos, São Paulo
**Problems**	**Poor knowledge and beliefs**<br>○ Dementia is a natural part of aging<br>○ Dementia only affects older people<br>○ Every person who lives with dementia was born with a predisposition to dementia<br>○ The person is to blame for the development of dementia (e.g., lifestyle)<br>○ The brain has not developed<br>○ Nothing can be done to support people living with dementia and their carers	**Stereotypes and labelling**<br><u>Visions that people living with dementia are:</u><br>○ Dependent and incapable<br>○ Passive<br>○ Depressed and fragile<br>○ Isolated and alone<br>○ Crazy and unpredictable<br>○ Aggressive<br>○ Difficult to deal with<br>○ Burdensome<br>○ Repetitive<br>○ Abnormal<br>○ Plaintiffs<br>○ Manipulative<br>○ Unable to have feelings	**Separation, blame, avoidance, loss of status, social exclusion**<br>○ The person is not allowed to make decisions and to engage in social\alone activities and is discouraged from maintaining social bonds and roles (e.g., work)<br>○ The person is no longer considered to be part of groups to which they were before dementia<br>○ The person has the value of their opinion and contribution reduced, and is ignored or avoided<br>○ The person is expected to obey and 'behave' and has their freedom removed and\or restricted<br>○ The person is blamed for the stigma and discrimination (e.g., excluded due to being considered repetitive)<br>○ The person suffers physical and emotional abuse<br>○ There is a reduction in social ties and participation<br>○ Carers feel isolated and excluded<br>○ Carers tend to keep the person living with dementia indoors to "protect" them		**Internalized stigma**<br>○ Low self-esteem<br>○ Doubt own feelings and experiences<br>○ Denial of dementia<br>○ Diagnosis concealment<br>○ Social withdrawn<br>○ Loss of identity<br>○ Reduced wellbeing	**Less access to health services**<br>**Poor quality services**<br>○ Lack of support services<br>○ Lack of training and qualified professionals<br>○ Reduced help seeking<br>○ Late diagnosis<br>○ Care mostly focused on physical needs<br>○ The care provided generates dependence and is passive<br>○ Care does not involve the visions and desires of the person living with dementia and their carers<br>○ Carers have high emotional and physical burden
**Intervention resources**	Audio-visual presentations	Video-narratives of people living with dementia and caregivers	Group discussions and reflections		Dosage: Six sessions, divided into three sequential meetings, lasting three hours per day (9h in all)	
**Activities**	What is dementia and how does it affect people living with dementia and their carers?	Experiences of people living with dementia and their carers.	Stigma, stereotypes, attributes, and language: discriminatory practices and their consequences.	Human rights-based practices, person-centred, and ethical care.	Strengthening tangible skills of compassion, empathy, and communication (verbal and nonverbal).	How can I improve my CHWs practice to better serve people living with dementia and their carers?<br>Brief and general evaluation of the programme
**Outcomes 30 days after intervention**	Improvement of knowledge and attitudes	Improved attitudes towards people living with dementia and their caregivers	Development of person-centred skills		Reduction of stigma levels	Reduction of discrimination levels

For peer review only - http://bmjopen.bmj.com/site/about/guidelines.xhtml

Baseline and post study assessment

Baseline (t0) and post study assessments (t2) will be the same for both experimental and control groups. Additional feasibility questions (closed and open questions) will be applied to the experimental group on the last day of the intervention as well as in t2, and 15 to 20 participants in the experimental group will be invited to participate in one-to-one semi-structured interviews. Apart from the qualitative interviews, the written questions are all self-administered and will be completed on paper. Questionnaires will be distributed and collected by an assistant researcher who will be blinded to participant allocation. We hope that using anonymous self-administered questionnaires (identifiable only though participant code) will help mitigate any potential risk for social desirability bias. We will also highlight to participants that the questionnaires are not identifiable and that any answers they provide will not be linked to their names, and that they should be free to answer honestly to all the questions without any fear of being judged. The researchers applying the intervention will not have access to the participants' responses until the end of data collection. It is expected that CHWs will take 10 to 15 minutes to complete all the questions. Considering the acceptance of approximately 14 PCUs to participate in the study, it is estimated that the total period between t0 and t2 in all PCUs will be 90 days.

Sociodemographic and stigma-related outcomes

Sociodemographic questions will include age, gender, level of education, religion, as well as information on previous training and experience with dementia (details in Supplementary material II). Stigma-related questions (dementia knowledge, attitudes and intended behaviour towards people living with dementia) were derived from the 2019 Global Stigma and Dementia Survey (WAR). The WAR questionnaire has been administered to more than 70,000 people worldwide including healthcare professionals, members of the public, people living with dementia, and their carers, through which process it has been translated to Brazilian Portuguese.[2] The WAR questions were designed in Likert scale format and are concerned with the dementia knowledge, attitudes and anticipated behaviour towards people living with dementia. The questions were informed by existing validated stigma and discrimination scales[35,36] and have been validated into a reduced number of items with high psychometric performance[2,37].

Feasibility assessment

First, a brief evaluation will be conducted on the last day of the intervention through discussion and notes will be taken by the researchers (e.g., relevance, positive and negative aspects). Then, in t2, additional measures (objective and subjective) regarding the feasibility of the intervention will be applied, including a measure of satisfaction with the intervention[38]

For peer review only - http://bmjopen.bmj.com/site/about/guidelines.xhtml

and open questions (details in Supplementary material II). We will also collate information on recruitment rate and retention overall and per session, intervention completion rate, evaluation measures completion, appropriateness/acceptability, and fidelity of the intervention. Provisional decision rules for what procedures to carry through to the full trial will include: retention of at least 70% completing at least two-thirds of the sessions, less than 15% missing on outcome measures and fidelity of at least 75% according to a fidelity checklist to be completed by a member of the research team in every session.[39–41] Qualitative and quantitative findings will be collated and reported in a transparent, and the decision regarding whether the intervention is feasible or not will be taken (plus justified and reported) upon discussion among the research team.

Nested qualitative component

Two participants from each PCU participating in the experimental group (n~14), including those who dropped out the study, will be invited to voluntarily participate in individual semi-structured interviews to explore further aspects of feasibility and potential impact of the intervention (details in Supplementary material III). Interviews will take place on the same day of t2, after all the post-study measures have been completed. The sample will be selected purposively, aiming to include a variety of sociodemographic characteristics, locations, and background training on dementia. To reduce the potential selection bias based on the interpersonal experience of the researchers applying the intervention with the participants, another member of the team will make this selection based on the characteristics reported in the questionnaires, and another researcher will conduct the interview itself. The interviews will last a maximum of 60 min, will be voice recorded, and will be held in a private room at the PCUs where the CHWs work.

Data analysis

We will seek to adhere to the steps proposed in the modelling phase of the Medical Research Council guidelines for developing complex interventions.[42] The purpose of a feasibility study is not to make a formal analysis of the primary outcome, but to evaluate trial processes to determine whether to progress to a study of effectiveness and to estimate parameters needed to design the future trial.[42] Data analysis will be conducted by a researcher who has not been involved with the intervention itself and who is blinded to the arm allocation. Double data entry and checking will be used to ensure accuracy.

We hypothesize that the intervention is feasible and might be able to improve knowledge, attitudes and intended behaviour in relation to dementia among CHWs. Analysis of quantitative data will be based on “intention to treat”, that is, data from all participants will be

For peer review only - http://bmjopen.bmj.com/site/about/guidelines.xhtml

included regardless of their withdrawal from the study or not. Descriptive analysis will include central and dispersion measures, according to the types (e.g., continuous, categorical, nominal) and distribution patterns of the variables, including number of incomplete responses and dropouts, ceiling, and floor effects. Differences in levels of variables related to dementia stigma, namely knowledge, attitudes and intended behaviours towards people living with dementia, before and after the intervention, between groups, and between time points (t0 and t2), will be analysed using Student's t test or a non-parametric test, according to data distribution. We will measure internal reliability (Cronbach's alpha) and test-retest, levels of variance, correlation between sociodemographic variables, and between the different outcome variables. An appropriate statistical approach will be chosen to handle any missing data depending on its pattern and type of variables.

Quantitative feasibility measures will be analysed descriptively and will be compared with the results from the outcome measures. Feasibility data collected through qualitative methods will be analysed in an integrated manner using content analysis as well as triangulation techniques in NVivo®.[43,44] In preparation to that, semi-structured interviews will first be transcribed anonymously and verbatim.

Ethics and dissemination

This study was approved by the National Commission for Ethics in Research in Brazil (CONEP) (*number removed for peer review*). Every individual taking part will be informed about their rights as participants, including the fact that nonparticipation will not affect in any way their work status or care received at the PCU. Every participant will sign a consent form; those participating in the semi structured interviews will be required to sign a second consent form specific for this research activity.

Safety monitoring procedures have been created to protect participants and researchers, including safety measures to prevent emotional impact and the spread of COVID-19 (e.g., mandatory vaccination and use of mask during any study activity, as well as physical distance during the study. All personal information about potential and enrolled participants will be collected, shared, and maintained in line with the norms and regulations of the Brazilian National Ethics Committee (http://www.conselho.saude.gov.br/comissoes-cns/conep) regarding confidentiality measures stated in order to protect confidentiality before, during, and after the study. Any important protocol modifications will be immediately communicated to the Research Ethics Committee and the study will be stopped until an approval is obtained for the study to continue. The study will not have a data monitoring committee (DMC) as this is not required for a feasibility non-pharmacological study in Brazil.

For peer review only - http://bmjopen.bmj.com/site/about/guidelines.xhtml

We plan to disseminate this study in open access scientific and community events related to dementia. The intervention materials will be published online and will be available for use by anyone who wishes to translate, adapt, and implement it. Our research team works closely with policy makers as part of a national advisory team, which we hope will help increase the possibility of such intervention to be applied in more settings in Brazil. An anti-stigma intervention toolkit will be informed by this study will be accessible nationally in Brazil and globally through open access publication to support researchers and practitioners with the implementation of dementia-related anti-stigma actions.

Patient and public involvement

Through a series of focus groups and semi-structured interviews, we have explored the views of family carers, people living with dementia, health care workers and members of the public about stigma and discrimination related to people living with dementia. The findings from this exploratory activity have informed the development of the intervention. We have also presented and discussed this intervention protocol among the international and multidisciplinary (*research programme name*) team for internal validation.[34] As this is a protocol for a feasibility study which involves both quantitative and qualitative procedures, we aim to gather participants' views about the intervention as part of the study itself in order to improve it for a future trial. We will also continue to consult our international consortium, which includes experts by experience, about the various methodological and practical aspects of the work as it progresses.

Planned study dates

We plan to start the study in August 2022 and to end it in January 2023.

Discussion

The number of people living with dementia in Brazil is likely to increase in the next decades, however the number of people who are undiagnosed remains high and there is limited access and availability of appropriate support. Improving knowledge, attitudes, and behaviour of healthcare workers towards people living with dementia is a global health priority. In doing so, this is likely to reduce the stigma and discrimination experienced by people living with the condition, protect the quality of their lives, improve diagnosis rates, and quality care. Anti-stigma interventions have been successful in other countries and disease contexts, such as other mental health conditions and HIV. However, there is a paucity of research on anti-stigma interventions related to dementia, particularly targeting health care workers.

For peer review only - http://bmjopen.bmj.com/site/about/guidelines.xhtml

For peer review only

To our knowledge, this is the first anti-stigma intervention related to dementia to be conducted in Brazil and we are not aware of any other similar intervention in other Latin American countries. We will target CHWs who are the first point of healthcare access for people living with dementia in the community and we hope this will help increase the number of people living with dementia who are identified and attended by the healthcare systems. As CHWs work alongside multidisciplinary teams, we hope that the learning experiences achieved through this intervention will be shared among other members of the team, contributing to a further reach of the intervention and higher impact. The measures of feasibility included in this protocol will be paramount to ensure that the invention is acceptable, relevant, and effective to be applied in a future randomized controlled trial in Brazil and in other LMICs. An anti-stigma toolkit will be created from the intervention will also help ensure fidelity of future trials, as well as applicability in PCUs whose teams have the opportunity to apply it in their territories.

Author contribution: DO has led the design and development of the intervention, and the writing of the manuscript; CG has supported with the development of the intervention and has contributed to the writing of the manuscript; FAFM, EM, ACF, NF, SEL, and CPF have supported with the design and development of the intervention and have contributed to the writing of the manuscript.

Abbreviations

CHWs: Community Health Workers

CONEP: National Commission for Ethics in Research

LMICs: Low- and Middle-Income Countries

PCU: Primary Healthcare Unit

Availability of data and materials

Reasonable requests for the datasets used and/or analysed during the current study will be considered by the study team. Model consent form is also available upon request.

Funding

This project is sponsored by UK Research and Innovation Global Challenges Research Fund (ES/P010938/1) (https://www.ukri.org/what-we-offer/collaborating-internationally/global-challenges-research-fund/). The study sponsor had no role in study design; collection, management, analysis, and interpretation of data; writing of the protocol;

For peer review only - http://bmjopen.bmj.com/site/about/guidelines.xhtml

and the decision to submit the protocol for publication, nor they had ultimate authority over any of these activities.

Competing interests

The authors declare no conflict of interest.

References

1. World Health Organization. Global status report on the public health response to dementia [Internet]. Geneva: World Health Organization; 2021 [cited 2021 Sep 9]. Available from: https://apps.who.int/iris/handle/10665/344701

2. Alzheimer Disease International. World Alzheimer Report 2019: Attitudes to dementia [Internet]. 2019 [cited 2020 May 11]. Available from: https://www.alz.co.uk/research/world-report-2019

3. Alzheimer Disease International. World Alzheimer Report 2021: Journey through the diagnosis of dementia [Internet]. 2021. Available from: (in press)

4. Link BG, Phelan J. Stigma power. Social Science & Medicine. 2014 Feb;103:24–32.

5. Zimmermann M. Alzheimer's Disease Metaphors as Mirror and Lens to the Stigma of Dementia. Lit Med. 2017;35(1):71–97.

6. Nguyen T, Li X. Understanding public-stigma and self-stigma in the context of dementia: A systematic review of the global literature. Dementia. 2020 Feb;19(2):148–81.

7. Rüsch N, Angermeyer MC, Corrigan PW. Mental illness stigma: Concepts, consequences, and initiatives to reduce stigma. Eur psychiatr. 2005 Dec;20(8):529–39.

8. Werner P. Stigma and Alzheimer's disease: A systematic review of evidence, theory, and methods. The stigma of disease and disability: Understanding causes and overcoming injustices. 2014;223–44.

9. Oliveira D, Da Mata FAF, Mateus E, Musyimi CW, Farina N, Ferri CP, et al. Experiences of stigma and discrimination among people living with dementia and family carers in Brazil: qualitative study. Ageing and Society. 2021 May 20;1–22.

10. Evans SC. Ageism and Dementia. In: Ayalon L, Tesch-Römer C, editors. Contemporary Perspectives on Ageism [Internet]. Cham: Springer International Publishing; 2018. p. 263–75. Available from: https://doi.org/10.1007/978-3-319-73820-8_16

11. Thornicroft G, Mehta N, Clement S, Evans-Lacko S, Doherty M, Rose D, et al. Evidence for effective interventions to reduce mental-health-related stigma and discrimination. The Lancet. 2016 Mar 12;387(10023):1123–32.

12. Thornicroft G, Brohan E, Rose D, Sartorius N, Leese M, INDIGO Study Group. Global pattern of experienced and anticipated discrimination against people with schizophrenia: a cross-sectional survey. Lancet. 2009 Jan 31;373(9661):408–15.

For peer review only - http://bmjopen.bmj.com/site/about/guidelines.xhtml

13. Semrau M, Evans-Lacko S, Koschorke M, Ashenafi L, Thornicroft G. Stigma and discrimination related to mental illness in low- and middle-income countries. Epidemiol Psychiatr Sci. 2015 Oct;24(5):382–94.

14. Lopez RP, Rose KM, Kenney L, Sanborn V, Davis JD. Managing Shame: A Grounded Theory of How Stigma Manifests in Families Living With Dementia. J Am Psychiatr Nurses Assoc. 2019;(9507418):1078390319832965.

15. Schmutte T, Olfson M, Maust DT, Xie M, Marcus SC. Suicide risk in first year after dementia diagnosis in older adults. Alzheimer's & Dementia. 2021 May 25;alz.12390.

16. Brossard B. Losing credibility. In: Forgetting Items: The Social Experience of Alzheimer's Disease [Internet]. Indiana University Press; 2017 [cited 2020 Aug 4]. p. 35–70. Available from: https://www.jstor.org/stable/j.ctvpb3z16.6

17. Hatzenbuehler ML. Structural stigma: Research evidence and implications for psychological science. American Psychologist. 2016 Nov;71(8):742–51.

18. Clay J, Eaton J, Gronholm PC, Semrau M, Votruba N. Core components of mental health stigma reduction interventions in low- and middle-income countries: a systematic review. Epidemiol Psychiatr Sci. 2020 Sep 4;29:e164.

19. Prins M, Veerbeek M, Willemse BM, Pot AM. Use and impact of the Alzheimer Experience: a free online media production to raise public awareness and enhance knowledge and understanding of dementia. Aging & Mental Health. 2019 Mar 5;1–8.

20. Farina N, Suemoto CK, Burton JK, Oliveira D, Frost R. Perceptions of dementia amongst the general public across Latin America: a systematic review. Aging & Mental Health. 2020 Feb 12;1–10.

21. Ibanez A, Flichtentrei D, Hesse E, Dottori M, Tomio A, Slachevsky A, et al. The power of knowledge about dementia in Latin America across health professionals working on aging. Alzheimer's & Dementia: Diagnosis, Assessment & Disease Monitoring [Internet]. 2020 Jan [cited 2020 Oct 20];12(1). Available from: https://onlinelibrary.wiley.com/doi/10.1002/dad2.12117

22. Nakamura AE, Opaleye D, Tani G, Ferri CP. Dementia underdiagnosis in Brazil. The Lancet. 2015 Jan;385(9966):418–9.

23. Fagundes SD, Silva MT, Thees MFRS, Pereira MG. Prevalence of dementia among elderly Brazilians: a systematic review. Sao Paulo Med J. 2011 Jan 6;129(1):46–50.

24. OPAS/PAHO. Mental Health [Internet]. 2020 [cited 2021 Jan 27]. Available from: https://www.paho.org/en/topics/mental-health

25. Patel V, Maj M, Flisher AJ, De Silva MJ, Koschorke M, Prince M, et al. Reducing the treatment gap for mental disorders: a WPA survey. World Psychiatry. 2010 Oct;9(3):169–76.

26. Oliveira D, et al. Stigma and discrimination related to people living with dementia among healthcare workers and members of the public in Brazil: Qualitative study. in preparation. n\d;

For peer review only - http://bmjopen.bmj.com/site/about/guidelines.xhtml

27. Chan AW, Tetzlaff JM, Gotzsche PC, Altman DG, Mann H, Berlin JA, et al. SPIRIT 2013 explanation and elaboration: guidance for protocols of clinical trials. BMJ. 2013 Jan 9;346(jan08 15):e7586–e7586.

28. Chan AW, Tetzlaff JM, Altman DG, Laupacis A, Gøtzsche PC, Krleža-Jerić K, et al. SPIRIT 2013 Statement: Defining Standard Protocol Items for Clinical Trials. Ann Intern Med. 2013 Feb 5;158(3):200.

29. World Health Organization. WHO QualityRights Tool Kit [Internet]. 2012 [cited 2021 Feb 4]. Available from: https://www.who.int/mental_health/publications/QualityRights_toolkit/en/

30. Brooker DJ, Latham I. Person-Centred dementia care: Making services better with the VIPS Framework. 2nd ed. London: Jessica Kingsley Publishers; 2016.

31. Edwards R, Voss S, Iliffe S. Education about dementia in primary care: is person-centredness the key?. DEMENTIA. 2014;13(1):111–9.

32. Kitwood T. Malignant social psychology. In: Understanding care, welfare and community a reader [Internet]. London; New York: Routledge; 2006 [cited 2021 Feb 3]. Available from: https://ebookcentral.proquest.com/lib/ulaval/detail.action?milDocID=11111

33. North J. Achieving Person-Centred Health Systems: Evidence, Strategies and Challenges [Internet]. 1st ed. Nolte E, Merkur S, Anell A, editors. Cambridge University Press; 2020 [cited 2020 Dec 28]. Available from: https://www.cambridge.org/core/product/identifier/9781108855464/type/book

34. Ghate D. Developing theories of change for social programmes: co-producing evidence-supported quality improvement. Palgrave Commun. 2018 Dec;4(1):90.

35. Bakolis I, Thornicroft G, Vitoratou S, Rüsch N, Bonetto C, Lasalvia A, et al. Development and validation of the DISCUS scale: A reliable short measure for assessing experienced discrimination in people with mental health problems on a global level. Schizophrenia Research. 2019 Oct;212:213–20.

36. Brohan E, Clement S, Rose D, Sartorius N, Slade M, Thornicroft G. Development and psychometric evaluation of the Discrimination and Stigma Scale (DISC). Psychiatry Research. 2013 Jun;208(1):33–40.

37. D'Amico F, Bhatt J, Comas-Herrera A, Farina N, Gaber S, Knapp M, et al. Development and validation of a knowledge scale for stigma in dementia using responses from the general public. (submmited for publication). 2022;

38. Wang Y, Xiao LD, Ullah S, He GP, De Bellis A. Evaluation of a nurse-led dementia education and knowledge translation programme in primary care: A cluster randomized controlled trial. Nurse Education Today. 2017 Feb;49:1–7.

39. Hoffmann TC, Glasziou PP, Boutron I, Milne R, Perera R, Moher D, et al. Better reporting of interventions: template for intervention description and replication (TIDieR) checklist and guide. BMJ. 2014 Mar 7;348(mar07 3):g1687–g1687.

40. Sangraula M, van't Hof E, Luitel NP, Turner EL, Marahatta K, Nakao JH, et al. Protocol for a feasibility study of group-based focused psychosocial support to improve the psychosocial well-being and functioning of adults affected by humanitarian crises in

For peer review only

Nepal: Group Problem Management Plus (PM+). Pilot Feasibility Stud. 2018 Dec;4(1):126.

41. Saunders RP, Evans MH, Joshi P. Developing a Process-Evaluation Plan for Assessing Health Promotion Program Implementation: A How-To Guide. Health Promotion Practice. 2005 Apr;6(2):134–47.

42. O'Cathain A, Croot L, Duncan E, Rousseau N, Sworn K, Turner KM, et al. Guidance on how to develop complex interventions to improve health and healthcare. BMJ Open. 2019 Aug;9(8):e029954.

43. Bengtsson M. How to plan and perform a qualitative study using content analysis. NursingPlus Open. 2016;2:8–14.

44. Erlingsson C, Brysiewicz P. A hands-on guide to doing content analysis. African Journal of Emergency Medicine. 2017 Sep 1;7(3):93–9.

For peer review only

For peer review only - http://bmjopen.bmj.com/site/about/guidelines.xhtml

Figure 2. Study flow

For peer review only

For peer review only - http://bmjopen.bmj.com/site/about/guidelines.xhtml

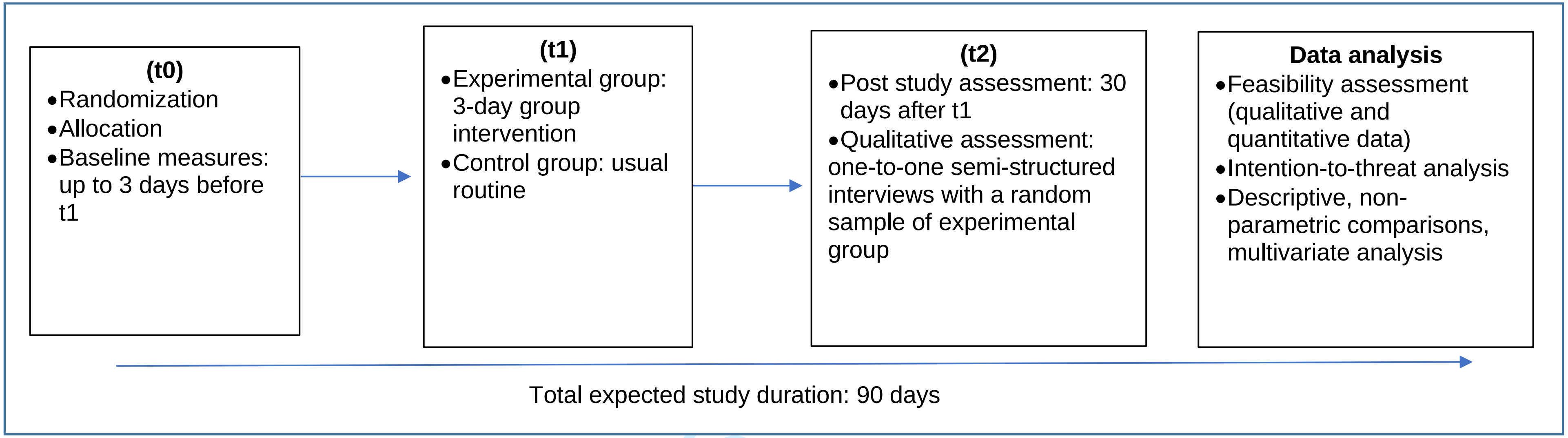

Figure 1. Study flow

review only

STANDARD PROTOCOL ITEMS: RECOMMENDATIONS FOR INTERVENTIONAL TRIALS

SUPPLEMMENTARY MATERIAL I

SPIRIT 2013 Checklist: Recommended items to address in a clinical trial protocol and related documents*

Section/item	Item No	Description	Addressed on page number
**Administrative information**			
Title	1	Descriptive title identifying the study design, population, interventions, and, if applicable, trial acronym	______1______
Trial registration	2a	Trial identifier and registry name. If not yet registered, name of intended registry	______5_______
	2b	All items from the World Health Organization Trial Registration Data Set	______5_______
Protocol version	3	Date and version identifier	______5_______
Funding	4	Sources and types of financial, material, and other support	______16______
Roles and responsibilities	5a	Names, affiliations, and roles of protocol contributors	______16______
	5b	Name and contact information for the trial sponsor	______16______
	5c	Role of study sponsor and funders, if any, in study design; collection, management, analysis, and interpretation of data; writing of the report; and the decision to submit the report for publication, including whether they will have ultimate authority over any of these activities	______16_____
	5d	Composition, roles, and responsibilities of the coordinating centre, steering committee, endpoint adjudication committee, data management team, and other individuals or groups overseeing the trial, if applicable (see Item 21a for data monitoring committee)	______NA_____

For peer review only - http://bmjopen.bmj.com/site/about/guidelines.xhtml

Introduction

Background and rationale	6a	Description of research question and justification for undertaking the trial, including summary of relevant studies (published and unpublished) examining benefits and harms for each intervention	______2-4____
	6b	Explanation for choice of comparators	______4______
Objectives	7	Specific objectives or hypotheses	______5______
Trial design	8	Description of trial design including type of trial (e.g., parallel group, crossover, factorial, single group), allocation ratio, and framework (e.g., superiority, equivalence, noninferiority, exploratory)	______5______

Methods: Participants, interventions, and outcomes

Study setting	9	Description of study settings (e.g., community clinic, academic hospital) and list of countries where data will be collected. Reference to where list of study sites can be obtained	_____5________
Eligibility criteria	10	Inclusion and exclusion criteria for participants. If applicable, eligibility criteria for study centres and individuals who will perform the interventions (e.g., surgeons, psychotherapists)	_____5_______
Interventions	11a	Interventions for each group with sufficient detail to allow replication, including how and when they will be administered	_____6-11_____
	11b	Criteria for discontinuing or modifying allocated interventions for a given trial participant (e.g., drug dose change in response to harms, participant request, or improving/worsening disease)	_____NA______
	11c	Strategies to improve adherence to intervention protocols, and any procedures for monitoring adherence (e.g., drug tablet return, laboratory tests)	______10_______
	11d	Relevant concomitant care and interventions that are permitted or prohibited during the trial	_____NA______
Outcomes	12	Primary, secondary, and other outcomes, including the specific measurement variable (e.g., systolic blood pressure), analysis metric (e.g., change from baseline, final value, time to event), method of aggregation (e.g., median, proportion), and time point for each outcome. Explanation of the clinical relevance of chosen efficacy and harm outcomes is strongly recommended	_12-13, Supp. 1__

For peer review only - http://bmjopen.bmj.com/site/about/guidelines.xhtml

Participant timeline	13	Time schedule of enrolment, interventions (including any run-ins and washouts), assessments, and visits for participants. A schematic diagram is highly recommended (see Figure)	12-13 and Fig. 1
Sample size	14	Estimated number of participants needed to achieve study objectives and how it was determined, including clinical and statistical assumptions supporting any sample size calculations	_____5_______
Recruitment	15	Strategies for achieving adequate participant enrolment to reach target sample size	_____10______

Methods: Assignment of interventions (for controlled trials)

Allocation:

Sequence generation	16a	Method of generating the allocation sequence (e.g., computer-generated random numbers), and list of any factors for stratification. To reduce predictability of a random sequence, details of any planned restriction (e.g., blocking) should be provided in a separate document that is unavailable to those who enrol participants or assign interventions	_____5-6______
Allocation concealment mechanism	16b	Mechanism of implementing the allocation sequence (e.g., central telephone; sequentially numbered, opaque, sealed envelopes), describing any steps to conceal the sequence until interventions are assigned	____5-6_______
Implementation	16c	Who will generate the allocation sequence, who will enrol participants, and who will assign participants to interventions	____5-6_______
Blinding (masking)	17a	Who will be blinded after assignment to interventions (e.g., trial participants, care providers, outcome assessors, data analysts), and how	__5-6, 12-14__
	17b	If blinded, circumstances under which unblinding is permissible, and procedure for revealing a participant's allocated intervention during the trial	____NA_______

Methods: Data collection, management, and analysis

Data collection methods	18a	Plans for assessment and collection of outcome, baseline, and other trial data, including any related processes to promote data quality (e.g., duplicate measurements, training of assessors) and a description of study instruments (e.g., questionnaires, laboratory tests) along with their reliability and validity, if known. Reference to where data collection forms can be found, if not in the protocol	____12-14_____

	18b	Plans to promote participant retention and complete follow-up, including list of any outcome data to be collected for participants who discontinue or deviate from intervention protocols	___12-13__
Data management	19	Plans for data entry, coding, security, and storage, including any related processes to promote data quality (e.g., double data entry; range checks for data values). Reference to where details of data management procedures can be found, if not in the protocol	______12____
Statistical methods	20a	Statistical methods for analysing primary and secondary outcomes. Reference to where other details of the statistical analysis plan can be found, if not in the protocol	______14____
	20b	Methods for any additional analyses (e.g., group and adjusted analyses)	_____14______
	20c	Definition of analysis population relating to protocol non-adherence (e.g., as randomised analysis), and any statistical methods to handle missing data (e.g., multiple imputation)	_____14______
**Methods: Monitoring**			
Data monitoring	21a	Composition of data monitoring committee (DMC); summary of its role and reporting structure; statement of whether it is independent from the sponsor and competing interests; and reference to where further details about its charter can be found, if not in the protocol. Alternatively, an explanation of why a DMC is not needed	____15______
	21b	Description of any interim analyses and stopping guidelines, including who will have access to these interim results and make the final decision to terminate the trial	____NA_______
Harms	22	Plans for collecting, assessing, reporting, and managing solicited and spontaneously reported adverse events and other unintended effects of trial interventions or trial conduct	_____15_____
Auditing	23	Frequency and procedures for auditing trial conduct, if any, and whether the process will be independent from investigators and the sponsor	_____14____
**Ethics and dissemination**			
Research ethics approval	24	Plans for seeking research ethics committee/institutional review board (REC/IRB) approval	_____14, 15___

For peer review only - http://bmjopen.bmj.com/site/about/guidelines.xhtml

Protocol amendments	25	Plans for communicating important protocol modifications (e.g., changes to eligibility criteria, outcomes, analyses) to relevant parties (e.g., investigators, REC/IRBs, trial participants, trial registries, journals, regulators)	______14_______
Consent or assent	26a	Who will obtain informed consent or assent from potential trial participants or authorised surrogates, and how (see Item 32)	_____6, 12______
	26b	Additional consent provisions for collection and use of participant data and biological specimens in ancillary studies, if applicable	______NA______
Confidentiality	27	How personal information about potential and enrolled participants will be collected, shared, and maintained in order to protect confidentiality before, during, and after the trial	_____13-14_____
Declaration of interests	28	Financial and other competing interests for principal investigators for the overall trial and each study site	______17_____
Access to data	29	Statement of who will have access to the final trial dataset, and disclosure of contractual agreements that limit such access for investigators	______15_____
Ancillary and post-trial care	30	Provisions, if any, for ancillary and post-trial care, and for compensation to those who suffer harm from trial participation	___14-15______
Dissemination policy	31a	Plans for investigators and sponsor to communicate trial results to participants, healthcare professionals, the public, and other relevant groups (eg, via publication, reporting in results databases, or other data sharing arrangements), including any publication restrictions	______15_____
	31b	Authorship eligibility guidelines and any intended use of professional writers	______16______
	31c	Plans, if any, for granting public access to the full protocol, participant-level dataset, and statistical code	______15______
**Appendices**			
Informed consent materials	32	Model consent form and other related documentation given to participants and authorised surrogates	______16_______
Biological specimens	33	Plans for collection, laboratory evaluation, and storage of biological specimens for genetic or molecular analysis in the current trial and for future use in ancillary studies, if applicable	______NA_____

For peer review only - http://bmjopen.bmj.com/site/about/guidelines.xhtml

*It is strongly recommended that this checklist be read in conjunction with the SPIRIT 2013 Explanation & Elaboration for important clarification on the items. Amendments to the protocol should be tracked and dated. The SPIRIT checklist is copyrighted by the SPIRIT Group under the Creative Commons "Attribution-NonCommercial-NoDerivs 3.0 Unported" license.

For peer review only

For peer review only - http://bmjopen.bmj.com/site/about/guidelines.xhtml

Supplementary material II

Baseline and post-study assessment

Sociodemographic and work-related data

1. Date of birth: _____________
2. Age: _______
3. Sex: Female ( ) Male ( ) Prefer not to say ( )
4. School attendance:

   Primary school ( ) Middle school ( ) Undergraduate degree or above ( )
5. Religion: None ( ) Catholic ( ) Evangelical ( ) Other: _________________ Prefer not to say ( )
6. Race: Black ( ) White ( ) Mixed-race ( ) Yellow ( ) Prefer not to say ( )
7. PCU name: ____
8. Number of months\years working as a CHW ___________________________
9. Do you have any <u>personal</u> experience working with people living with dementia or carers?

   Yes ( ) No ( ) Does not know or cannot remember ( )
10. Do you have any <u>professional</u> experience working with people living with dementia or carers?

    Yes ( ) No ( ) Does not know ( ) Cannot remember ( )
11. Have you ever had any training on dementia?

    Yes ( ) No ( ) Does not know ( ) Cannot remember ( )
12. If you said yes to the question above, when was this training and what was the focus?

    Month and year: __________________________

    Topic: ____

Global Survey on Stigma and Dementia (2019)

Questions on knowledge, attitudes and intended behaviours related to people living with dementia. Items available upon request: https://www.alzint.org/resource/world-alzheimer-report-2019/

Feasibility

Satisfaction with the intervention (Wang et al., 2017)

For peer review only

Instruction: Please read each of the items below and circle the answer that best reflects your opinion. We are trying to improve the intervention you participated in, so please be as honest as possible with your answers so we can do better in the future.

Questions	Strongly disagree	Disagree	I neither agree nor disagree	Agree	Strongly agree
1. Before participating in the program, my knowledge about dementia and caring for people living dementia was very limited.	1	2	3	4	5
2. This program has helped me learn new knowledge on dementia and care for people living with dementia.	1	2	3	4	5
3. The activities were necessary and useful.	1	2	3	4	5
4. The discussions improved my learning in relation to people living with dementia.	1	2	3	4	5
5. Discussions on how to translate knowledge into practice helped me think about how to apply knowledge in my practice.	1	2	3	4	5
6. The materials were clear and informative.	1	2	3	4	5
7. Overall, the program has reached my expectations.	1	2	3	4	5
8. Overall, I believe the program will help improve my work with people living with dementia and their carers.	1	2	3	4	5

For peer review only

Open questions

Please answer the questions below with your opinions about your participation in this project. We are trying to improve the intervention you participated in, so please be as honest as possible with your answers so we can do better in the future.

1. Would you recommend this program for another CHW? Why?

2. Overall, from 0 to 10, how satisfied were you with your participation in the program? Why? (0- no satisfied at all, 10- very satisfied)

3. What did you like the most in this programme? Why?

4. What did you not like in this programme? Why?

5. Was there anything that got in the way of your participation? If so, what was it?

6. What\How could the program be improved?

7. Have you talked about any of the aspects discussed in this programme with anyone?

8. How do you think participation in this program may have affected or will affect your practice as CHW?

9. Could you give a concrete example of any change that has already occurred in your CHW's activities in relation to people living with dementia and their families after your participation in the program? If you believe there has been no change, answer "no."

For peer review only

Supplementary material III

Interview schedule

Introduction

1. Thank the participant for taking part

2. The purpose of the interview

I'm part of a project called STRiDE. The idea of the project is …

We would like to hear your ideas and suggestions related to your participation in this project, so I'm going to ask you a few questions about this. I have here a list of questions and topics that will serve as a general guide on what I would like to talk to you about today, but this is flexible since the most important thing is that we talk about what is important to you.

The duration of the interview depends on your availability and interest. It usually lasts 60 min, but we can use more or less time. You can stop the interview at any time if you don't want to continue. It's okay if you want to take a break when you feel the need.

3. Reminder of ethical issues related to consent, anonymity, confidentiality, and safety measures

4. Presentations

5. Questions

Is there anything else you'd like to know about your participation or any other questions you may have?

6. Interview

1. Why did you decide to participate in this study?
2. What do you remember the most of what we covered in the sessions?
3. How would you describe the training and key messages covered?
4. Could tell me about any learning points you had through this programme?
5. What did you like the most and what did you like the least about this program?
6. Do you think the duration of the sessions and the whole study were sufficient or should be shorter or longer? Why is that?
7. If you could participate again, what would you like to be different?
8. What do you think we could do to get as many CHWs as possible involved in activities like this?
9. Do you consider that your participation in the programme is likely to change your practice as a CHW with people living with dementia and their carers in any way? Why\How?
10. Do you think participating in this programme and applying what you've learned in practice can help improve the care provided for people living with dementia and their carers in any way in real life? Why\How?
11. In your opinion, is there anything you think would limit how much you can put in practice what you learned during the intervention? Could you explain why?

For peer review only

12. In relation to the previous question, how could we researchers mitigate such factors?

13. Would you recommend this program to someone? Why is that?

14. Do you have any other comments to make about the intervention?

End of interview

1. Remind the participant about the end of the interview.
2. Thank the participant for their valuable contributions.
3. Tell the participant what will happen now (e.g. how the data will be used).

For peer review only
